# Author Correction: Proteomic profiling and genome-wide mapping of O-GlcNAc chromatin-associated proteins reveal an O-GlcNAc-regulated genotoxic stress response

**DOI:** 10.1038/s41467-021-26886-5

**Published:** 2021-11-16

**Authors:** Yubo Liu, Qiushi Chen, Nana Zhang, Keren Zhang, Tongyi Dou, Yu Cao, Yimin Liu, Kun Li, Xinya Hao, Xueqin Xie, Wenli Li, Yan Ren, Jianing Zhang

**Affiliations:** 1 School of Life and Pharmaceutical Sciences, Dalian University of Technology, Panjin, China; 2 Clinical Laboratory of BGI Health, BGI-Shenzhen, Shenzhen, China

**Keywords:** Glycobiology, Gene expression profiling, Stress signalling, Proteomics, Chromatin

Correction to: *Nature Communications* 10.1038/s41467-020-19579-y, published online 19 November 2020.

The original version of this Article incorrectly described and discussed the protein NRF1 as “nuclear factor erythroid 2-related factor-1”, while the correct full name of the NRF1 protein studied in this Article is “nuclear respiratory factor 1”. Any inference regarding nuclear erythroid 2-related factor-1 have been removed and do not otherwise affect the reported results. The PDF and HTML version of the Article as well as Supplementary Data 16 have been corrected to reflect this change.

## Supplementary information

Updated Supplementary Dataset 16

