# Supplementary material for: Proteomic profiling and genome-wide mapping of O-GlcNAc chromatin-associated proteins reveal an O-GlcNAc-regulated genotoxic stress response
Source: Nat Commun. 2020 Nov 19;11:5898. doi: 10.1038/s41467-020-19579-y (PMC7678849; doi:10.1038/s41467-020-19579-y)
Supplement: Supplementary file 1 — Supplementary Information [file 41467_2020_19579_MOESM1_ESM.pdf]

Supplementary information

Proteomic profiling and genome-wide mapping of O-GlcNAc chromatin-associated proteins reveal an O-GlcNAc-regulated genotoxic stress response

Yubo Liu[1, ¶], Qiushi Chen[2, ¶], Nana Zhang[1], Keren Zhang[2], Tongyi Dou[1], Yu Cao[1], Yimin Liu[1], Kun Li[1], Xinya Hao[1], Xueqin Xie[1], Wenli Li[1], Yan Ren[2, *], Jianing Zhang[1, *]

[1,] School of Life and Pharmaceutical Sciences, Dalian University of Technology, Panjin, China.

[2,] Clinical Laboratory of BGI Health, BGI-Shenzhen, Shenzhen, China.

Supplementary Figure 1

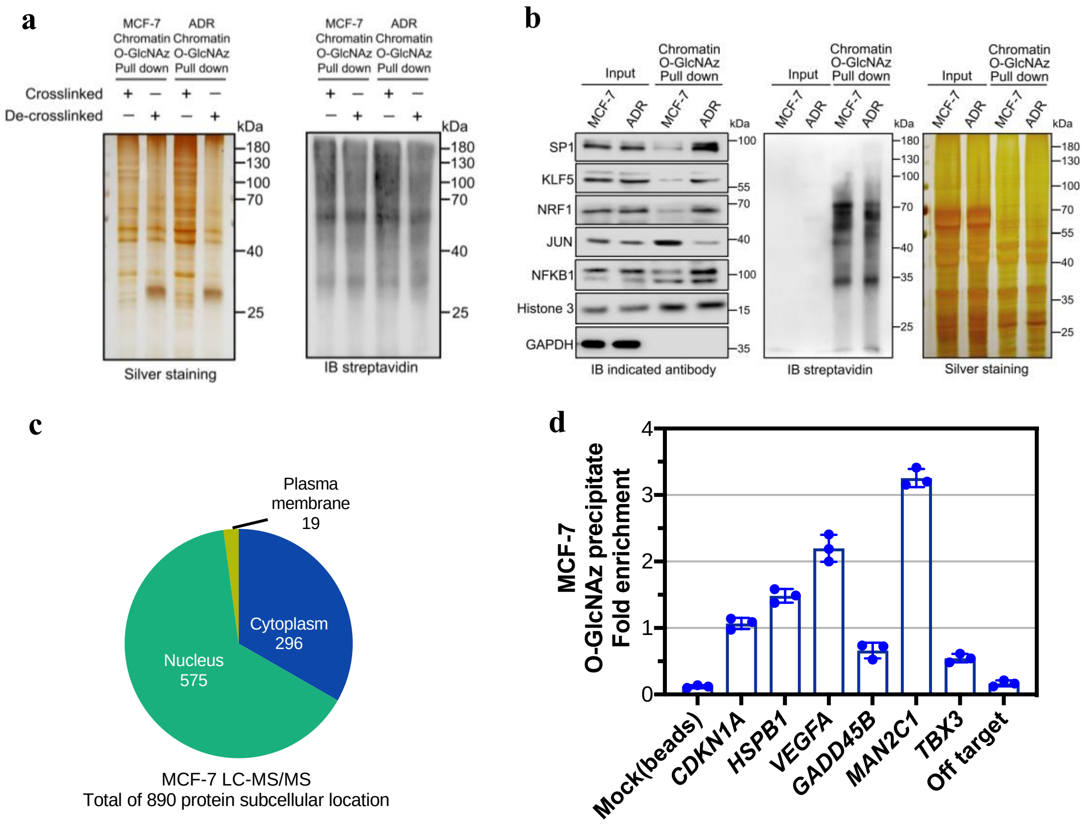

Supplementary Figure 1

OCPs are involved in the cellular stress response in MCF-7 cells. (**a**) Silver staining and immunoblotting showed the ability of 2% SDS to reverse the formaldehyde crosslinking of chromatin. MCF-7 and ADR cells were incubated with GalNAz and then subjected to formaldehyde crosslinking. The crosslinked chromatin was isolated, fragmented and reacted with alkyne-biotin. Chromatin was decrosslinked in 2% SDS buffer at 100°C for 20 min and then at 60°C for 2 h. The OCPs were then enriched with streptavidin-beads and strictly washed. The resulting OCPs on the beads were analyzed by immunoblotting and silver staining. After decrosslinking, the nonspecific contamination caused by PPIs between OCPs and other proteins was distinctly reduced. All blots and sliver staining are representative of at least two biologically independent experiments. Source Data are provided as a Source Data file. (**b**) Immunoblotting and silver staining of GalNAz-labeled MCF-7 and ADR cell lysates and chromatin. The chromatin proteins of the cells in the presence of GalNAz (24 h) were extracted, reacted

with alkyne-biotin, enriched with streptavidin beads and strictly wash. Immunoblotting was used to analyze the total cellular O-GlcNAc and chromatin binding of the indicated OCTFs in MCF-7 and ADR cells. All blots and silver staining are representative of at least two biologically independent experiments. Source Data are provided as a Source Data file. (**c**) The pie chart illustrates the subcellular localization of LC-MS/MS-identified O-GlcNAz proteins from MCF-7 chromatin. A total of 890 proteins in 990 proteins identified by LC-MS/MS with high confidence could be annotated. n = 9 biologically independent experiments. (**d**) The promoter regions of genotoxic stress-related genes can be enriched in the O-GlcNAz chromatin precipitate. Six indicated genes were selected for validation by ChIP-qPCR. Chromatin of GalNAz-labeled MCF-7 cells was extracted and reacted with alkyne-biotin. O-GlcNAz-binding DNA was subsequently decrosslinked and enriched for qPCR. qPCR amplification was performed using primers specific for the indicated gene promoters. Each bar represents the fold enrichment of the bound proteins relative to the input. Beads without streptavidin (mock) and random primers that could not specifically bind the indicated gene promoter regions (off target) were used as negative controls. The data are presented as the means ± SEM., n = 3 biologically independent experiments. Experiments were repeated independently two times with similar results. Source Data are provided as a Source Data file.

Supplementary Figure 2

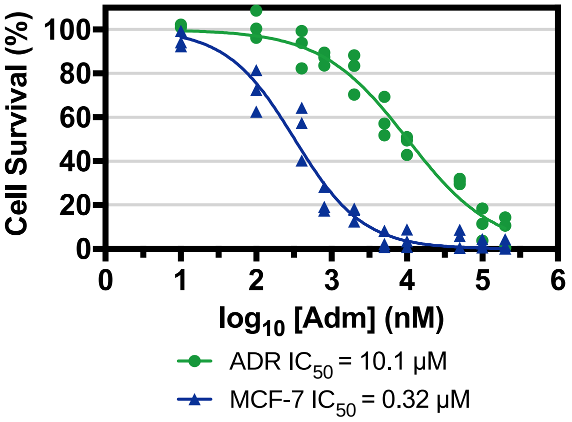

Supplementary Figure 2

MCF-7 and ADR cells were treated with increasing doses of Adm for 48 h and the cell viability was then assessed though a CCK8 assay. The IC_{50} values were calculated. Replicates are represented, n = 3 biologically independent experiments. Experiments were repeated independently two times with similar results. Source Data are provided as a Source Data file.

Supplementary Figure 3

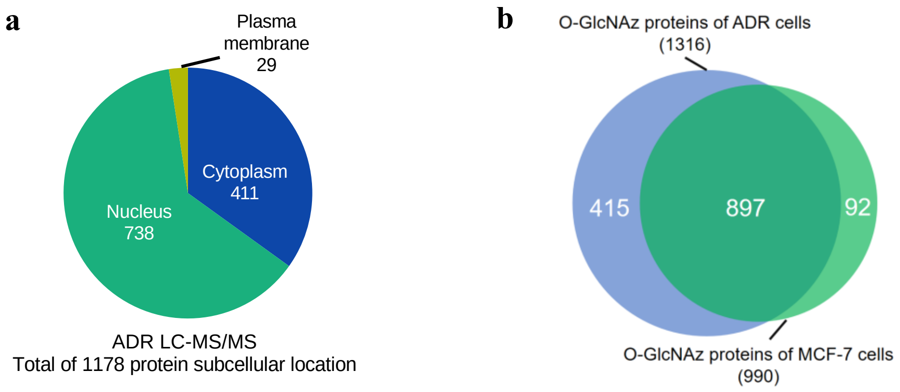

Supplementary Figure 3

LC-MS/MS analysis of O-GlcNAz proteins in MCF-7 and ADR cells. (**a**) The pie chart illustrates the subcellular localization of LC-MS/MS-identified O-GlcNAz proteins from ADR chromatin. A total of 1178 proteins in 1316 proteins identified by LC-MS/MS with high confidence could be annotated. n = 9 biologically independent experiments (**b**) Venn diagram showing the overlapping O-GlcNAz proteins identified in MCF-7 and ADR chromatin.

Supplementary Figure 4

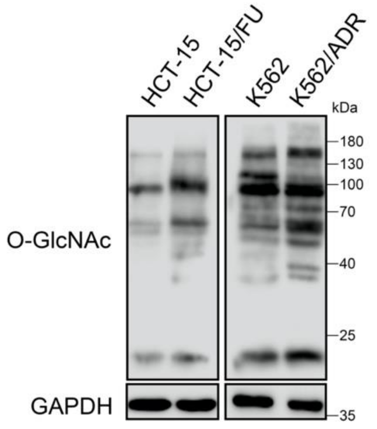

Supplementary Figure 4

Genotoxic stress-adapted cells displayed an increase in cellular O-GlcNAc modification compared with the parental cells. Human colon cancer cell line HCT-15 and the fluorouracil (FU) adapted cells HCT-15/FU, human chronic myeloid leukemia cell line K562 and the Adm adapted cells K562/ADR were analyzed for the total cellular O-GlcNAc with CTD110.6 antibodies. All blots are representative of at least two biologically independent experiments. Source Data are provided as a Source Data file.

Supplementary Figure 5

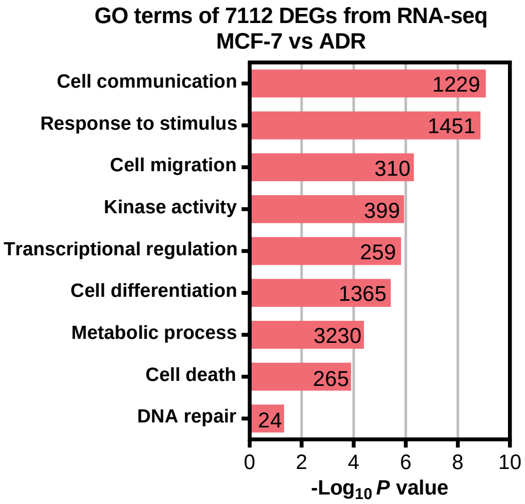

Supplementary Figure 5

GO terms enriched for RNA-seq DEGs in MCF-7 and ADR cells. A large number of DEGs are categorized into response to stimulus, cell death and DNA repair groups. The gene numbers identified in certain terms are shown. Source Data are provided as a Source Data file.

Supplementary Figure 6

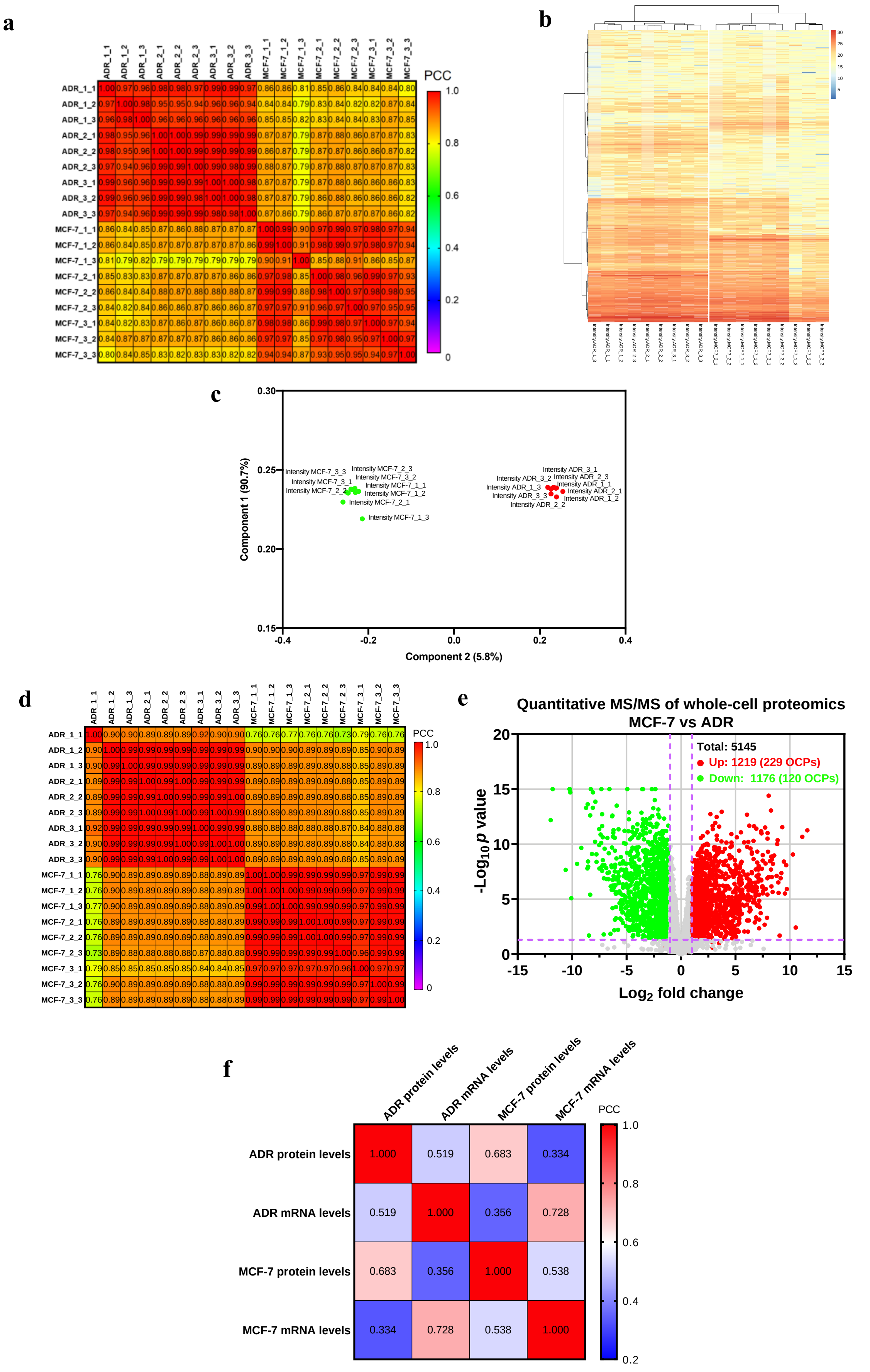

Supplementary Figure 6

Quantitative proteomics analysis of O-GlcNAz proteins in MCF-7 and ADR cells. (**a**) Correlation between nine biological replicates of chromatin O-GlcNAz protein quantitative proteomics analyses of MCF-7 and ADR cells. A heat map was generated by global Pearson correlation analysis. The Pearson correlation coefficient (PCC) is indicated. n = 9 biologically independent experiments. (**b**) Log_2 ratios of all the protein intensities were used to generate a heat map by hierarchical clustering. A heat map showed a high correlation between the biological replicates. n = 9 biologically independent experiments. (**c**) A PCA plot showed clear clustering between the MCF-7 versus ADR replicate samples. n = 9 biologically independent experiments. Source Data are provided as a Source Data file. (**d**) Correlation between nine biological replicates of whole-cell quantitative proteomics analyses of MCF-7 and ADR cells. A heat map was generated by global Pearson correlation analysis. The PCC is indicated. n = 9 biologically independent experiments. (**e**) Volcano plot of quantitative proteomics data from whole-cell proteins in MCF-7 and ADR cells. Proteins were considered significantly deregulated if the following criteria were met: fold change ≥ 2, p value ≤ 0.05 (two-sided unpaired Student's t-test), and FDR ≤ 0.05. A total of 5145 proteins were identified with high confidence (identified at least six times in nine biological replicates). A total of 1219 proteins (including 229 OCPs) and 1176 protein (including 120 OCPs) were upregulated and downregulated, respectively, in ADR cells compared with MCF-7 cells. n = 9 biologically independent experiments. (**f**) Correlation between whole-cell quantitative proteomics and related gene mRNA levels (RNA-seq) in MCF-7 and ADR cells. A heat map was generated by global Pearson correlation analysis. The PCC is indicated. The genes identified from both the whole-cell quantitative proteomics and RNA-seq analyses showed positive correlations with PCC > 0.5 in both MCF-7 and ADR cells. n = 2 biologically independent RNA-seq replicates. n = 9 biologically independent quantitative proteomics experiments.

Supplementary Figure 7

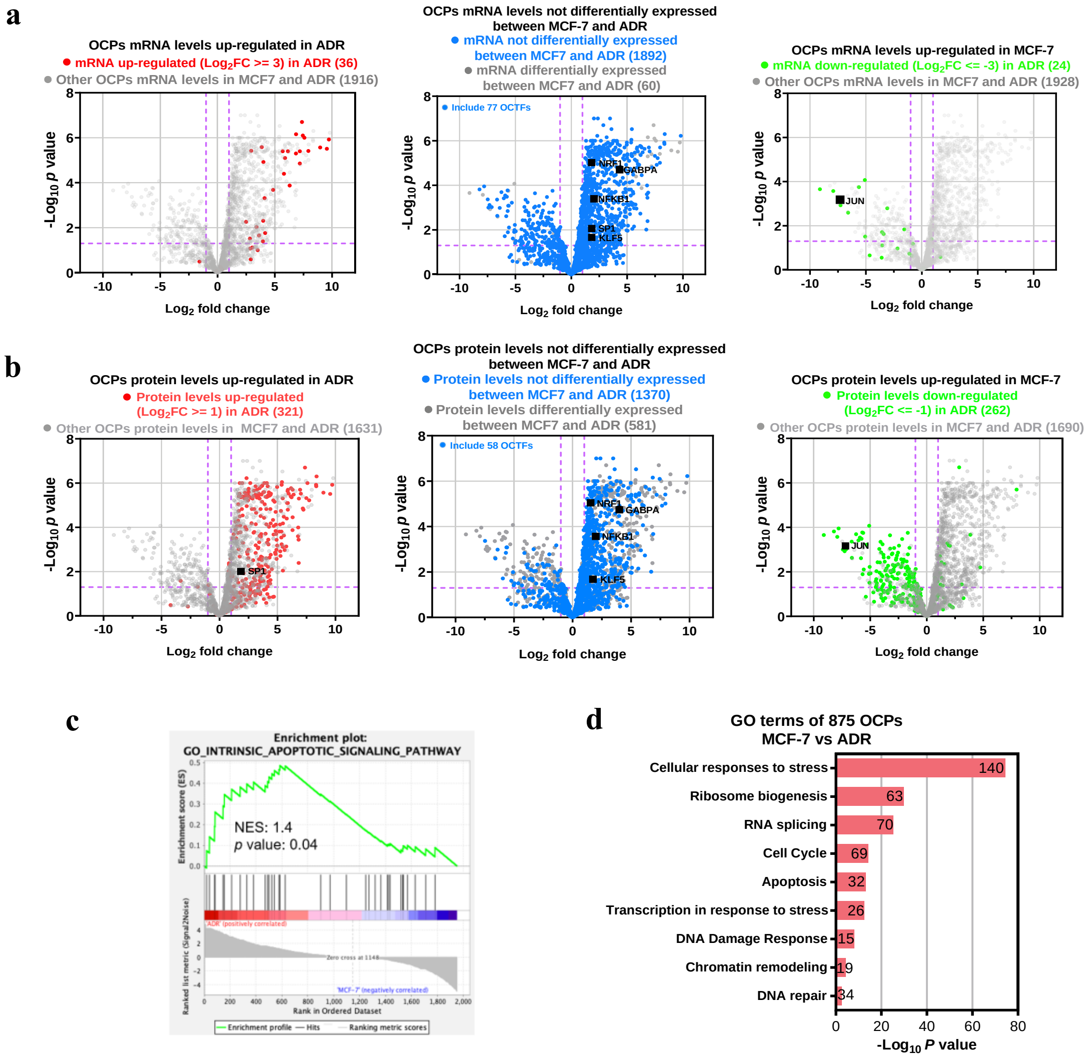

Supplementary Figure 7

Analysis of OCPs in MCF-7 and ADR cells. (**a**) A series of volcano plots of quantitative proteomics data of OCPs in MCF-7 and ADR cells. The OCPs are divided into three bins according to FPKM fold change of the corresponding genes obtained by RNA-seq: "OCPs mRNA levels upregulated in ADR" (Log_2 fold change (FC) ≥ 3), "OCPs mRNA levels not differentially expressed between MCF-7 and ADR" ($3 > Log_2$ FC > -3) and "OCPs mRNA levels upregulate in MCF-7" (Log_2 FC ≤ -3). Invisible transcriptional differences were found in OCPs identified by LC-MS/MS. The OCPs mentioned in this study are labeled. (**b**) The OCPs identified by quantitative proteomics were divided into three bins according to the corresponding fold change in protein level (protein must be identified in six out of nine replicates of at least one group, Log_2 FC ≤ -1 or ≥ 1, p value

$\leq$ 0.05, FDR $\leq$ 0.05) by whole-cell quantitative proteomics without O-GlcNAz enrichment: "OCP protein levels upregulated in ADR", "OCP protein levels not differentially expressed between MCF-7 and ADR" and "OCP protein levels upregulated in MCF-7". The protein levels of most OCPs were not changed between the two cells. (**c**) GSEA of OCPs revealing their enrichment in the pathways related to apoptosis in MCF-7 cells compared with ADR cells. NES and *p* value are indicated. (**d**) The GO analysis of 875 differential quantitative OCPs. The gene numbers identified in the certain terms are shown. Source Data are provided as a Source Data file. For (**a**) and (**b**), nine biologically independent quantitative proteomics experiments and two biologically independent RNA-seq replicates were performed.

Supplementary Figure 8

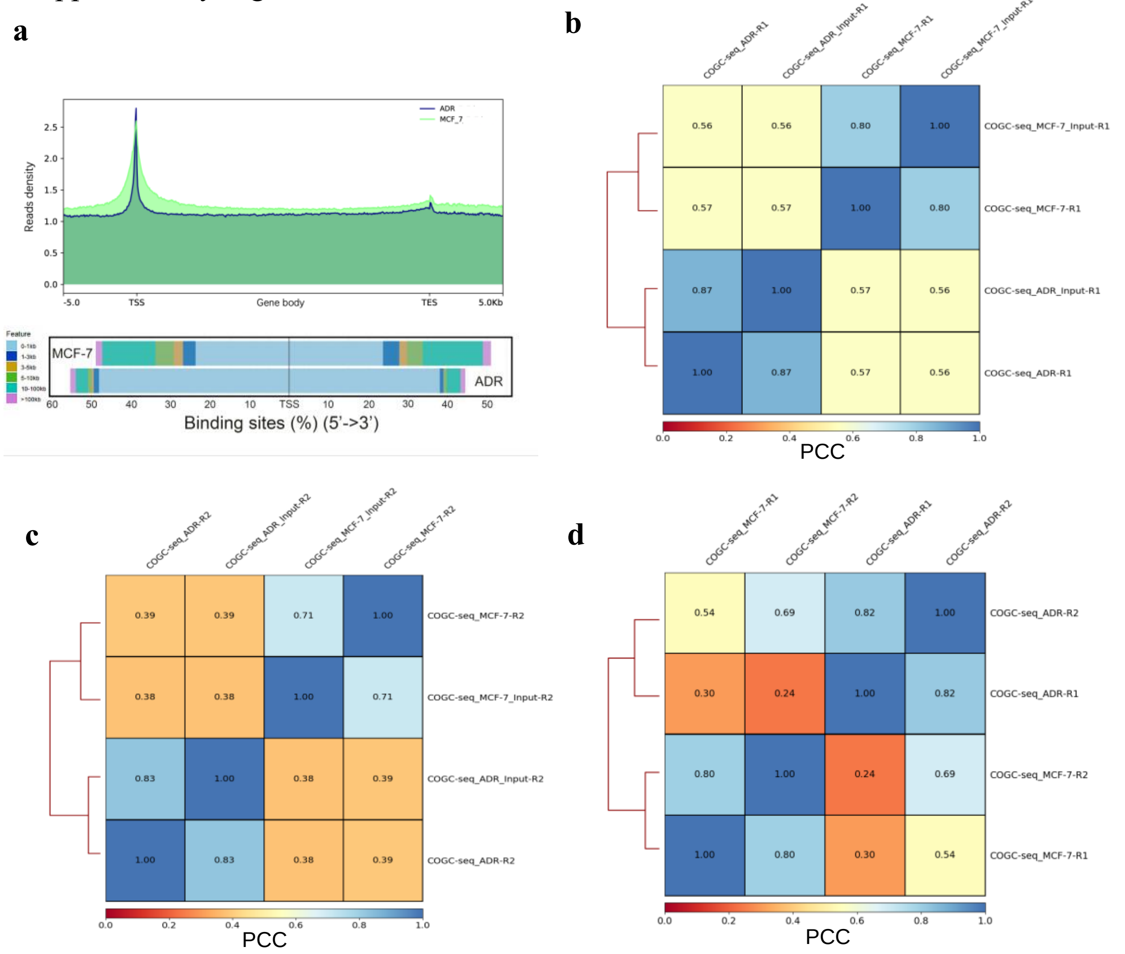

Supplementary Figure 8

Bioinformatics analysis of the MCF-7 and ADR COGC-seq datasets. (**a**) The upper image shows the average enrichment profiles of COGC-seq reads at all gene regions in the reference genome hg19. The TSS and termination site (TES) are indicated. The lower image shows the distribution of MCF-7 and ADR COGC-seq peaks relative to TSSs. ChIPseeker was used to calculate the percentage of peaks upstream and downstream from the TSSs of the nearest genes. (**b**) and (**c**) Correlation between COGC-seq and input datasets in MCF-7 and ADR cells. Two biological repetitive tests (R1 and R2) are indicated. (**d**) Correlation between two biological replicates of COGC-seq tests of MCF-7 and ADR cells. (**b-d**) Heat maps were generated by global Pearson correlation analysis of genome-wide reads from each COGC-seq bam file. The PCC is indicated.

Supplementary Figure 9

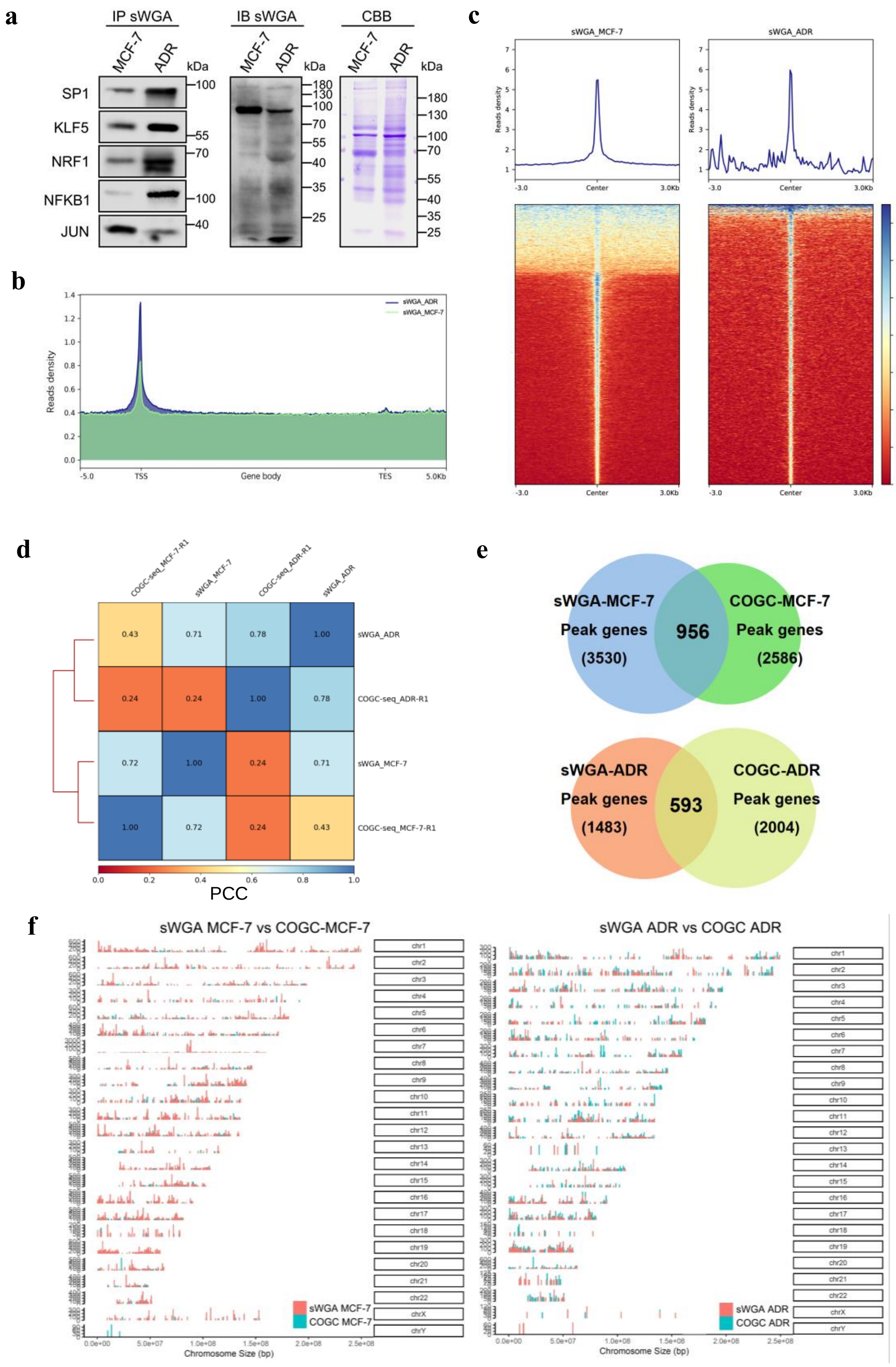

Supplementary Figure 9

Analysis of sWGA lectin ChIP-seq datasets in MCF-7 and ADR cells. (**a**) sWGA lectin blots and sWGA lectin ChIP of OCPs in MCF-7 and ADR cells. The crosslinked

chromatin of the indicated cells were extracted and immunoprecipitated using sWGA-agarose. Decrosslinked OCPs was analyzed by sWGA lectin blots and immunoblotting using indicated antibodies. Coomassie blue staining (CBB) of sWGA-agarose immunoprecipitated fractions was used as a control. Genotoxic stress increases the interactions of OCTFs (stress response-related SP1, NFKB1, KLF5, NRF1 and JUN) with chromatin. All blots and CBB are representative of at least two biologically independent experiments. Source Data are provided as a Source Data file. (**b**) The average enrichment profiles of sWGA lectin ChIP-seq reads at all gene regions in the reference genome hg19. The TSS and TES are indicated. Both MCF-7 and ADR signals specifically distributed at transcription start sites (TSSs) with a sharp single peak. sWGA lectin ChIP-seq was performed once. (**c**) Heat maps of sWGA lectin ChIP-seq signal density at peaks center (± 3 kb). The average signal profile is shown. The red color indicates low a signal, and a blue color indicates a high signal. (**d**) Correlation between COGC-seq and sWGA lectin ChIP-seq tests of MCF-7 and ADR cells. Heat maps were generated by global Pearson correlation analysis of genome-wide reads from each bam file. The PCC is indicated. COGC-seq and sWGA lectin ChIP-seq show strong correlation in MCF-7 and ADR cells, respectively. (**e**) Venn diagram showing the overlapping peak associated genes identified by sWGA lectin ChIP-seq and COGC-seq in the indicated cells. (**f**) Coverage plots of sWGA lectin ChIP-seq and COGC-seq peak locations over the whole human genome in the indicated cells. The results show that these two methods identified similar O-GlcNAc chromatin binding pattern.

Supplementary Figure 10

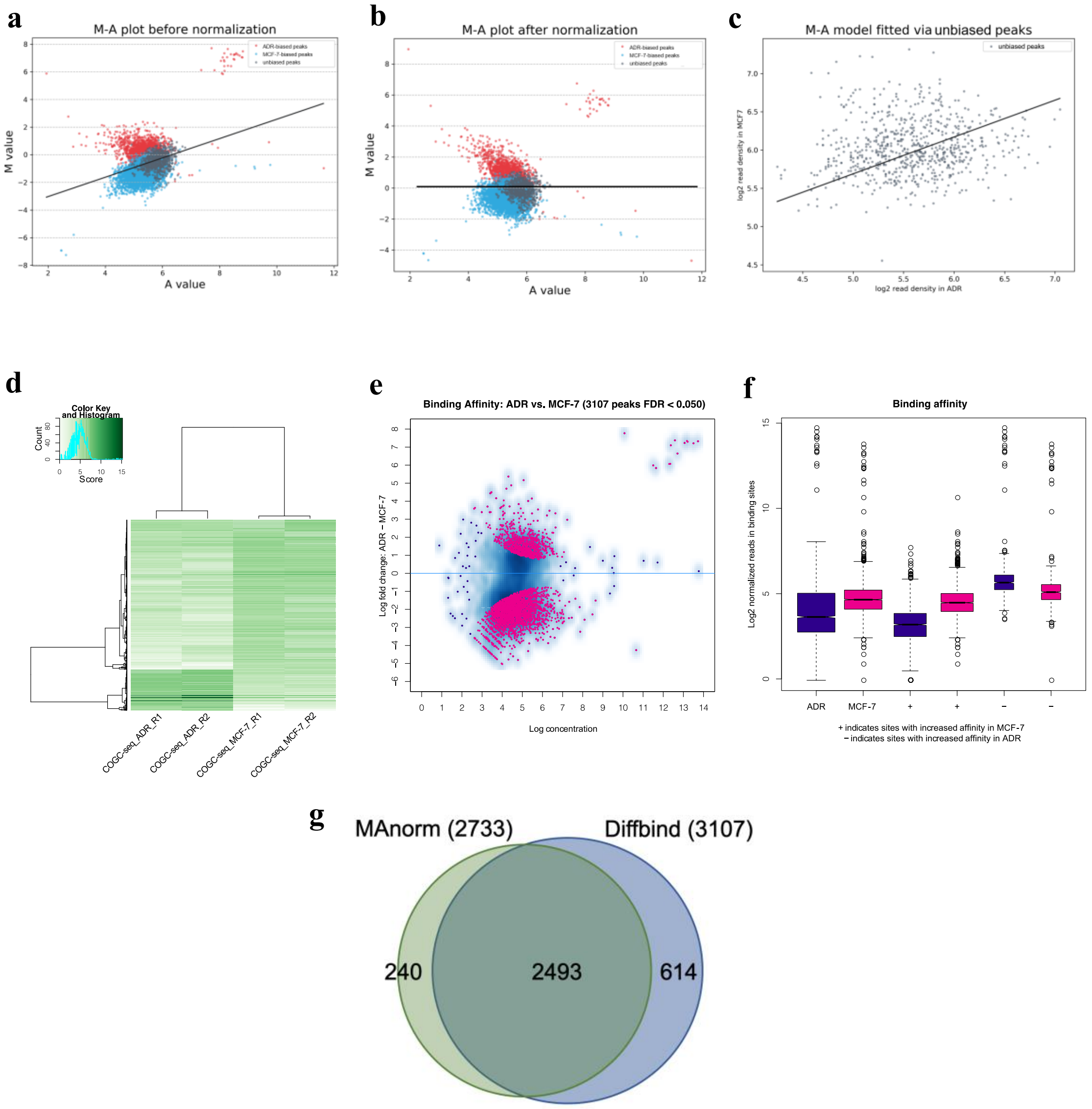

Supplementary Figure 10

Quantitative comparisons of COGC-seq peaks. (a-c) Normalization and quantitative comparisons of COGC-seq peaks using MAnorm. MA plots of all peaks from both MCF-7 and ADR COGC-seq datasets before (**a**) and after MAnorm (**b**). Differential quantitative regions ("ADR-biased" or "MCF-7-biased", fold change ≥ 2 and P value $\leq 10^{-5}$ for MAnorm). The line shows the linear model derived from "unbiased" peaks by robust regression. (**d-f**) Normalization and quantitative comparisons of COGC-seq peaks using Diffbind. Heatmap and hierarchical clustering representation of the differential quantitative (FDR $\leq$ 0.05) peaks (**d**). MA plot of resistant-responsive contrast using DiffBind, and the differential quantitative peaks are shown in red (**e**).

Box plots of read distributions obtained for the differential quantitative peaks (**f**). The box plots show the medians (black lines), 25th and 75th percentiles (boundaries), and minimum/maximum (whiskers). n = 2 biologically independent COGC-seq replicates. (**g**) Venn diagram showing the overlapping differential peaks identified by MAnorm and DiffBind. The differential binding regions determined by MAnorm and DiffBind were highly consistent.

Supplementary Figure 11

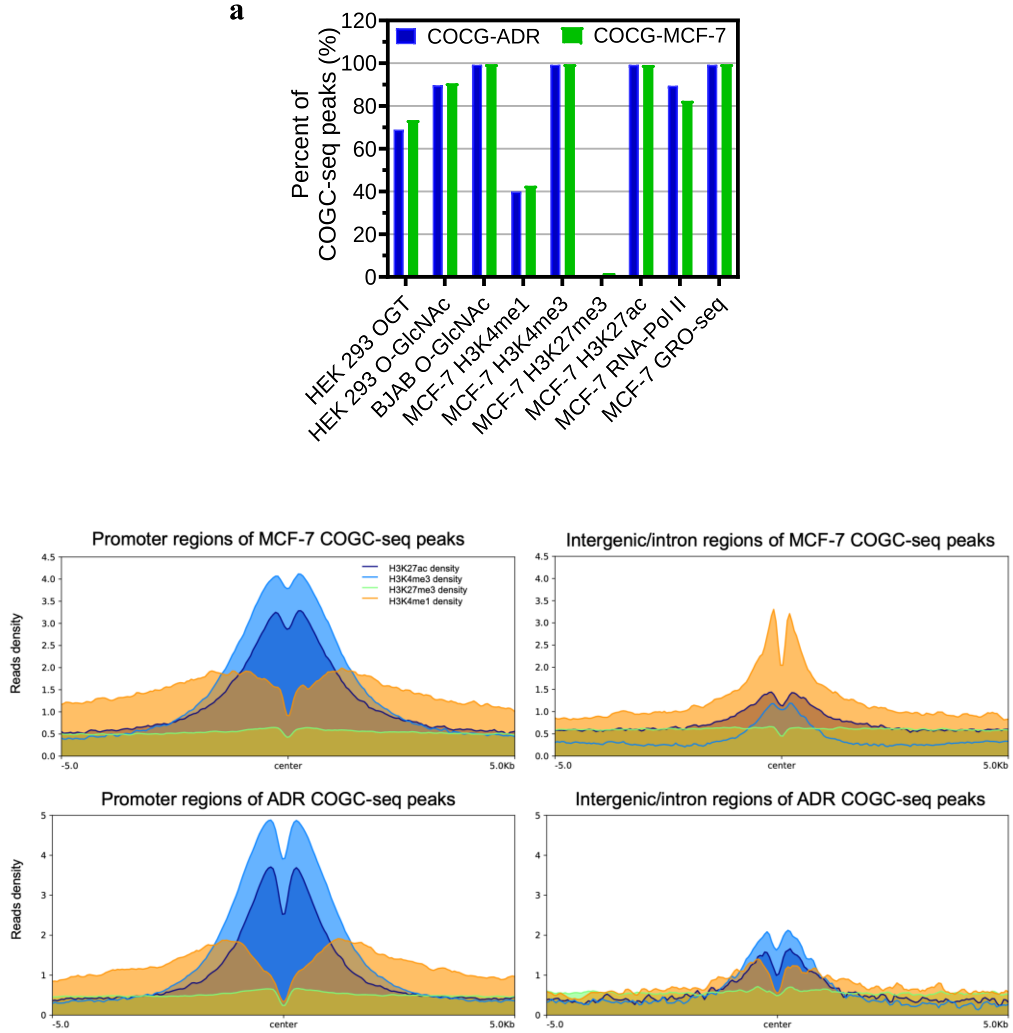

Supplementary Figure 11

COGC-seq peaks overlap with transcriptionally activated regions. (**a**) COGC-seq peaks overlapped significantly with previously reported OGT and O-GlcNAc ChIP-seq datasets, including HEK293 cells OGT, O-GlcNAc (GSE36620 [https://www.ncbi.nlm.nih.gov/geo/query/acc.cgi?acc=GSE36620]), and BJAB cells (Burkitt's lymphoma cell line) O-GlcNAc (GSE86154 [https://www.ncbi.nlm.nih.gov/geo/query/acc.cgi?acc=GSE86154]). Furthermore, COGC-seq peaks overlapped with the activated histone marks (published MCF-7 cells H3K4me1 (GSE86714 [https://www.ncbi.nlm.nih.gov/geo/query/acc.cgi?acc=GSE86714]), H3K4me3 and

H3K27ac, (GSE97481 [https://www.ncbi.nlm.nih.gov/geo/query/acc.cgi?acc=GSE97481]) ChIP-seq datasets), but almost no overlap was found with a marker of transcriptionally inactive chromatin (published MCF-7 cells H3K27me3 (GSE96363 [https://www.ncbi.nlm.nih.gov/geo/query/acc.cgi?acc=GSE96363]) ChIP-seq datasets). Almost all the O-GlcNAz peaks overlapped with transcriptionally activated regions (published MCF-7 cells RNA-Pol II ChIP-seq (GSE34001 [https://www.ncbi.nlm.nih.gov/geo/query/acc.cgi?acc=GSE34001]) and MCF-7 cells GRO-seq (GSE96859 [https://www.ncbi.nlm.nih.gov/geo/query/acc.cgi?acc=GSE96859]) datasets). Source Data are provided as a Source Data file. (**b**) Average enrichment profiles of above published H3K27ac, H3K4me3, H3K27me3 and H3K4me1 ChIP-seq reads density at indicated COGC-seq peaks.

Supplementary Figure 12

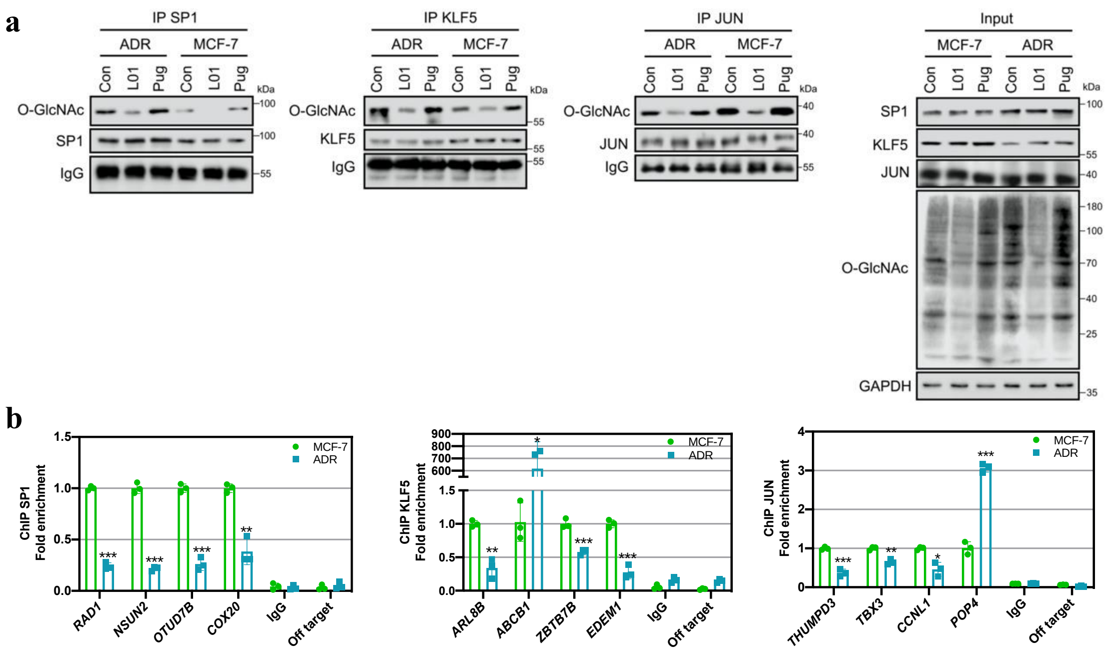

Supplementary Figure 12

Certain OCTF targeting genes were validated by ChIP-qPCR. (**a**) The O-GlcNAc modification of SP1, KLF5 and JUN was validated using the O-GlcNAc inhibitor L01 and agonist PugNAc. MCF-7 and ADR cells were treated with 100 µM L01 or PugNAc. DMSO was used as a control (Con). The indicated TF co-IP was performed, and immunoprecipitated fractions were analyzed by immunoblotting for the O-GlcNAc levels (CTD110.6). All blots are representative of at least two biologically independent experiments. Source Data are provided as a Source Data file. (**b**) The binding of SP1, KLF5 and JUN to downstream target genes was confirmed by ChIP-qPCR. ChIP assays were performed using the indicated TFs antibodies, and qPCR amplification was performed using primers specific for the target gene promoter. Each bar represents the fold enrichment of bound protein relative to the input. IgG and random primers that could not specifically bind the indicated gene promoter regions (off target) were used as negative controls. The data are presented as the means ± SEM., n = 3 biologically independent experiments, *$p < 0.05$, **$p < 0.01$, ***$p < 0.001$ (two-sided unpaired Student's t-test). p values: 0.000005, 0.000018, 0.000071, 0.001383 (ChIP SP1); 0.001757, 0.008195, 0.000872, 0.000381 (ChIP KLF5); 0.000394, 0.001614, 0.006923, 0.000053 (ChIP JUN). Experiments were repeated independently two times with

similar results. Source Data are provided as a Source Data file.

Supplementary Figure 13

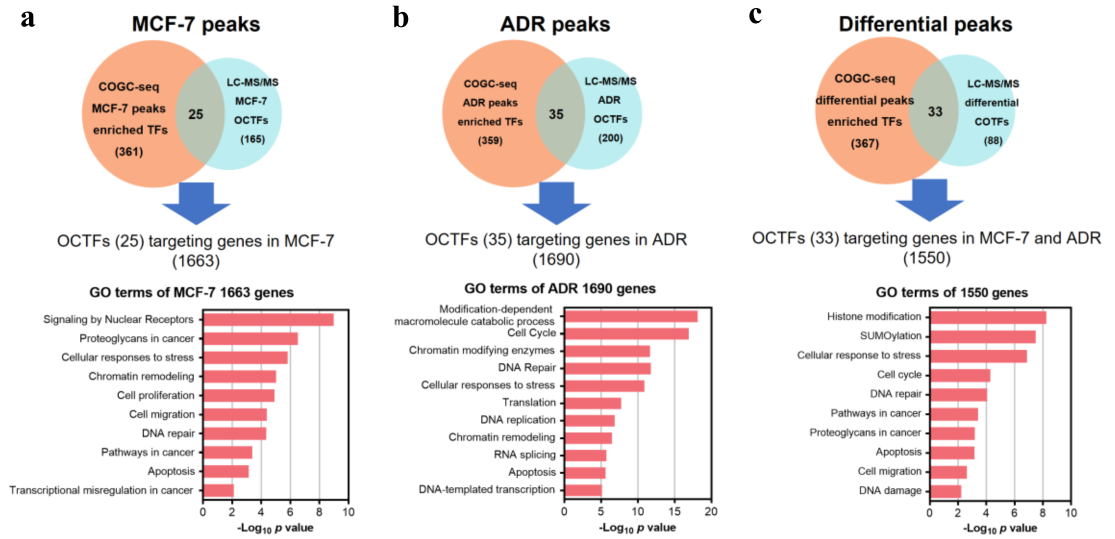

Supplementary Figure 13

OCTFs targeting genes are categorized for GO terms related to genotoxic stress. (**a-c**) Upper images show the overlap of TFs identified by COGC-seq motif enrichment analysis with OCTFs identified from LC-MS/MS. The overlapping OCTF-targeting genes were used for GO term analysis (lower images). These genes are categorized into stress response and DNA repair groups. Source Data are provided as a Source Data file.

Supplementary Figure 14

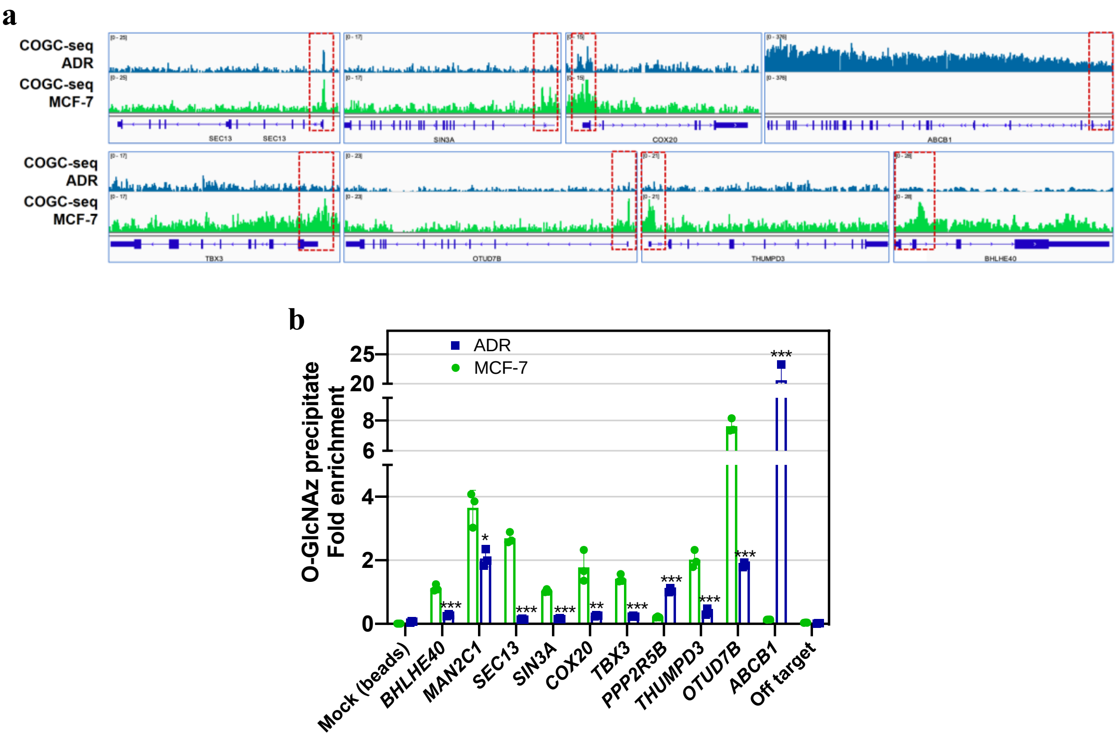

Supplementary Figure 14

Validation of OCTFs binding change in MCF-7 and ADR cells. (**a**) IGV tracks showing COGC-seq signal at the promoter region of the representative genes *SEC13*, *SIN3A*, *COX20*, *ABCB1*, *TBX3*, *OTUD7B*, *THUMPD3* and *BHLHE40*. The change of O-GlcNAz binding at promoter regions were observed during genotoxic stress response. (**b**) Ten genotoxic stress-related genes (mentioned in this study) were chosen for validating by ChIP-qPCR. The chromatin of GalNAz-labeled cells were extracted and click reacted with alkyne-biotin. O-GlcNAz binding DNA was subsequently decrosslinked and enriched for qPCR. qPCR amplification was performed using primers specific for the indicated gene promoters. Each bar represents the fold enrichment of binding relative to the input. Beads without streptavidin pull down (mock) and random primers that could not specifically bind the indicated genes promoter region (off target) were used as negative controls. The data are presented as the means ± SEM., n = 3 biologically independent experiments, *$p < 0.05$, **$p < 0.01$, ***$p < 0.001$ (two-sided unpaired Student's t-test). p values: 0.000173, 0.010894, 0.000014, 0.000009, 0.005978, 0.000094, 0.000021, 0.000613, 0.000026, 0.000112. Experiments were

repeated independently two times with similar results. Source Data are provided as a Source Data file.

Supplementary Figure 15

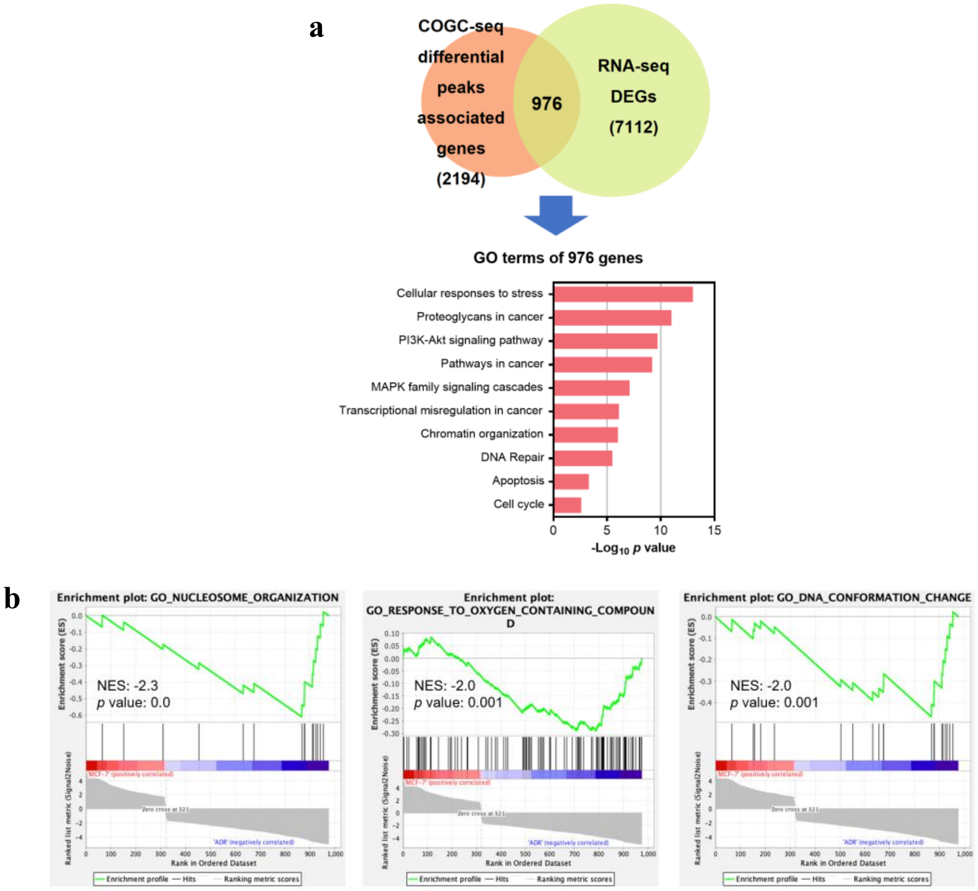

Supplementary Figure 15

O-GlcNAz associated genes are predominantly involved in cellular stress response and chromatin conformation change. (**a**) RNA-seq DEGs of MCF-7 and ADR cells are compared with COGC-seq differential peak associated genes. The overlapping 976 genes are used for the GO terms analysis. Source Data are provided as a Source Data file. (**b**) GSEA of above 976 genes revealing enrichment in the pathways related to cellular stress response and chromatin conformation change. NES and *p* value are indicated.

Supplementary Figure 16

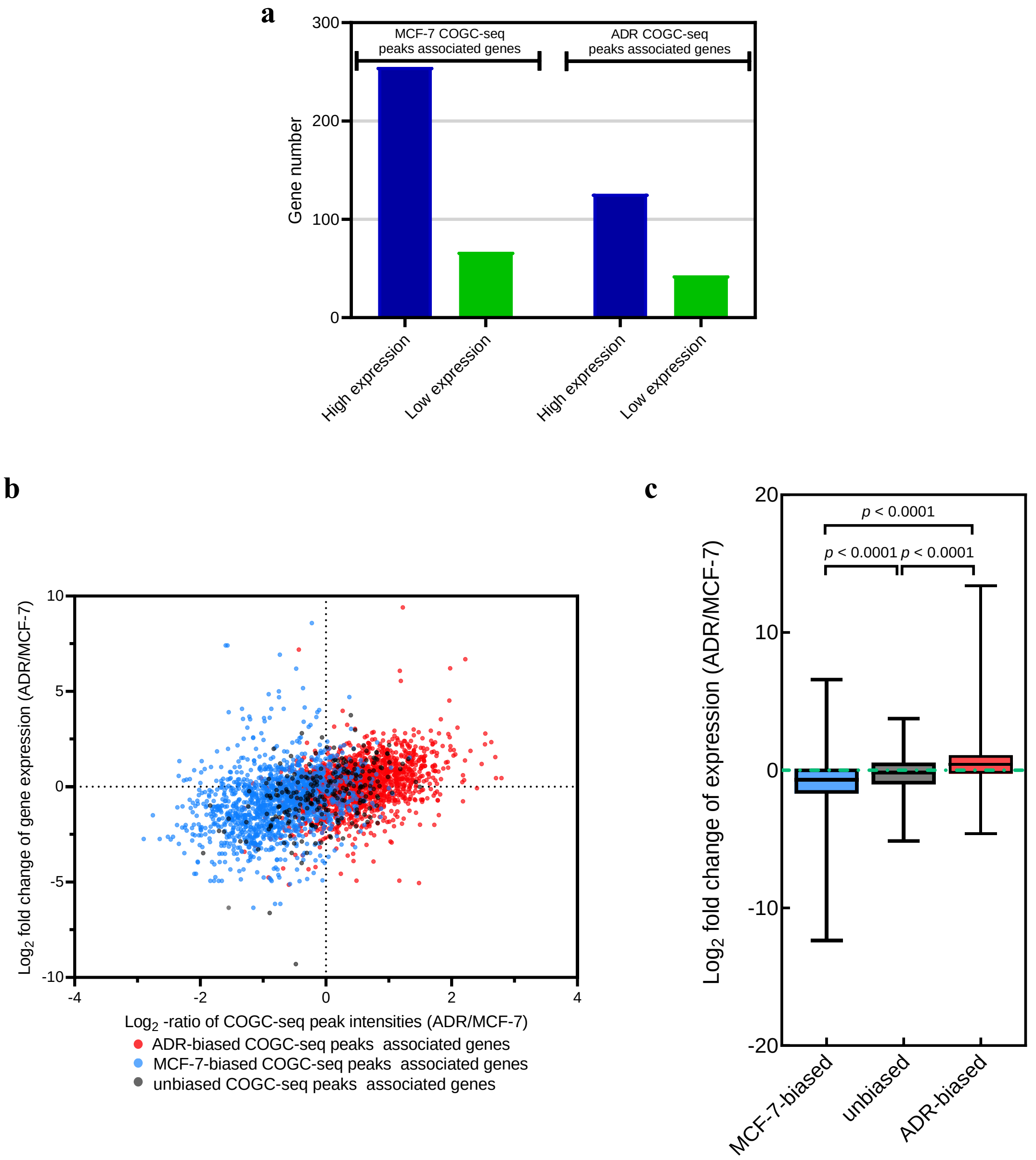

Supplementary Figure 16

Expression analysis of COGC-seq peak associated genes. (**a**) The expression of the majority of COGC-seq peak associated genes (OCTF-occupied genes) was upregulated during the genotoxic stress response. The bars show the number of upregulated (high expression, MCF-7 versus ADR, RNA-seq FPKM fold change ≥ 2) and downregulated (low expression, MCF-7 versus ADR, RNA-seq FPKM fold change ≤ 0.5) COGC-seq peak-associated genes in MCF-7 and ADR cells, respectively. Source Data are provided as a Source Data file. (**b**) Scatter plot of the Log_2-ratios of the gene expression levels (RNA-seq FPKM) between MCF-7 and ADR cells versus the Log_2-ratios of the COGC-

seq peak intensities (MAnorm M value) over all the COGC-seq peak associated genes in MCF-7 and ADR cells. Unbiased, MCF-7-biased and ADR-biased COGC-seq peak associated genes were plotted in different colors. For (**a**) and (**b**), two biologically independent COGC-seq and RNA-seq experiments were performed. (**c**) mRNA expression change (RNA-seq FPKM) of unbiased, MCF-7-biaesed and ADR-biased COGC-seq peak associated genes. The box plots show the medians (black lines), 25th and 75th percentiles (boundaries), and minimum/maximum (whiskers). Two biologically independent RNA-seq replicates were used. The *p* value (1.2E-15 (MCF-7-baised vs. unbaised), 2.4E-15 (unbaised vs. ADR-baised), 1.5E-15 (MCF-7-baised vs. ADR-baised), two-sided unpaired Student's t-test, calculated between multiple genes in each group) is indicated. n = 2 biologically independent RNA-seq replicates.

Supplementary Figure 17

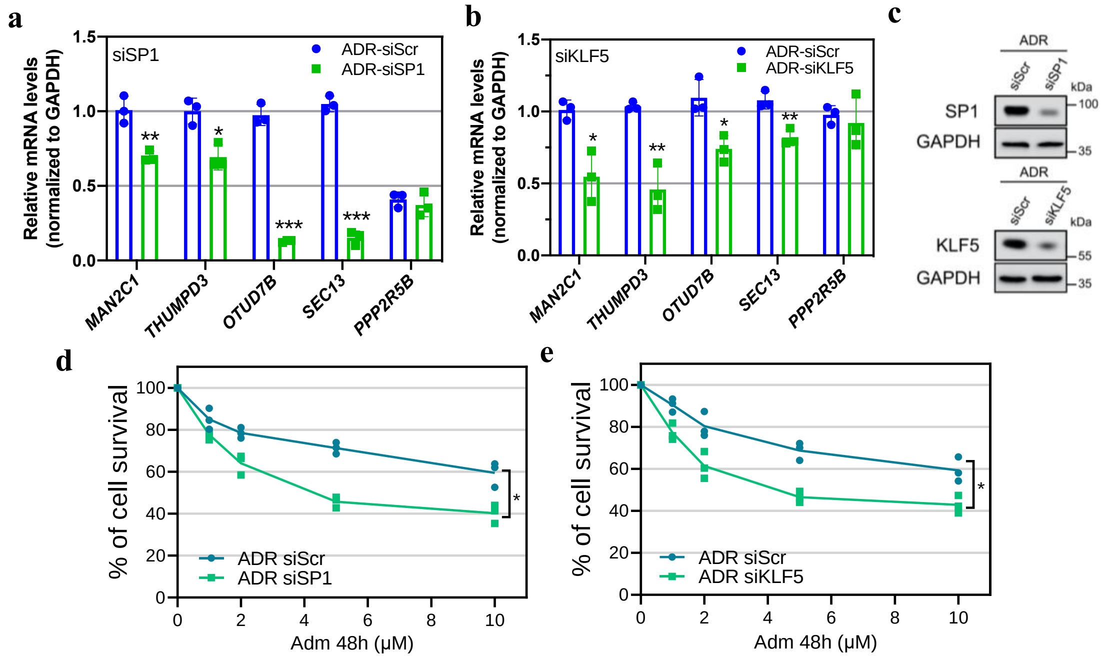

Supplementary Figure 17

The loss of OCTFs SP1 and KLF5 decrease downstream genes expression. (**a**-**b**) ADR cells were transfected with SP1 or KLF5 siRNA. The corresponding downstream genes (*MAN2C1*, *THUMPD3*, *OTUD7B*, *SEC13*, *PPP2R5B*) mRNA levels were analyzed by qPCR. Scrambled siRNA (siScr) was used as a control. The data are presented as the means ± SEM., n = 3 biologically independent experiments, *$p < 0.05$, **$p < 0.01$, ***$p < 0.001$ (two-sided unpaired Student's t-test). p values: 0.005677, 0.011653, 0.000032, 0.000023 (**a**); 0.013165, 0.003937, 0.016954, 0.005792 (**b**). Experiments were repeated independently two times (**a**-**b**) with similar results. (**c**) The protein levels of SP1 and KLF5 were analyzed by immunoblotting. All blots are representative of at least two biologically independent experiments. (**d**-**e**) ADR cells transfected with indicated siRNA were treated with increasing doses of Adm for 48 h. The cell viability was assessed using CCK8 assay. Replicates are represented, n = 3 biologically independent experiments, * $p < 0.05$ (two-sided unpaired Student's t-test). p values: 0.000002 (**d**); 0.000125 (**e**). Experiments were repeated independently two times (**d**) and (**e**) with similar results. (**a**-**e**) Source Data are provided as a Source Data file.

Supplementary Figure 18

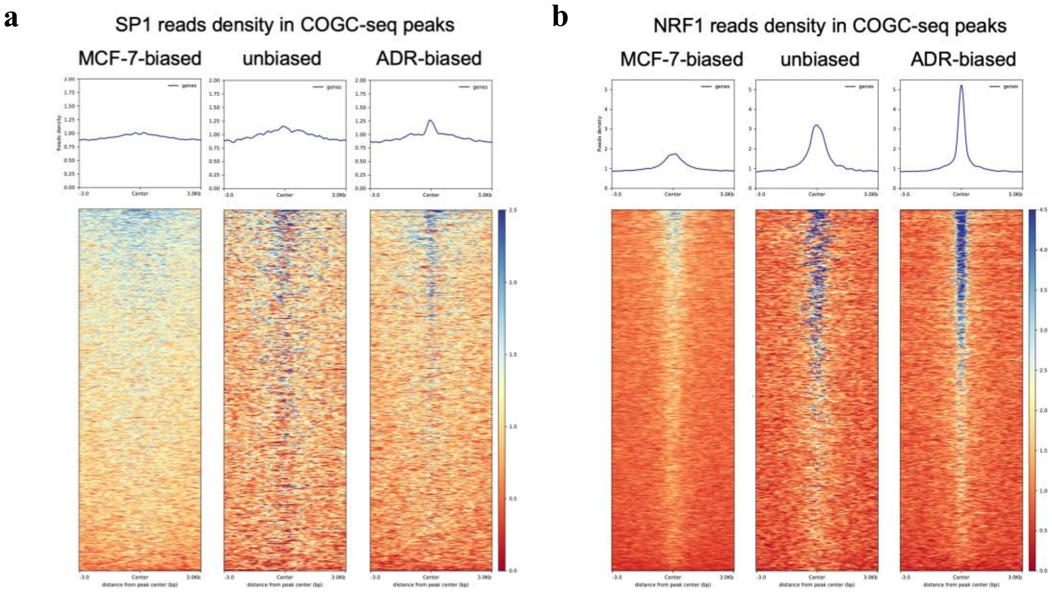

Supplementary Figure 18

Heat maps representation of the published (**a**) MCF-7 cells SP1 (GSE92014 [https://www.ncbi.nlm.nih.gov/geo/query/acc.cgi?acc=GSE92014]) and (**b**) NRF1 (GSE91522 [https://www.ncbi.nlm.nih.gov/geo/query/acc.cgi?acc=GSE91522]) ChIP-seq signal enrichment (red, low; blue, high) at MCF-7-biased, ADR-biased and unbiased COGC-seq peaks. Enrichment levels were profiled ± 3 kb from the peaks center. The average signal profile is shown. SP1 ChIP-seq signal showed similar levels across differential COGC-seq peaks, whereas substantial changes of NRF1 ChIP-seq signal distribution in MCF-7 and ADR-biased peaks were detected.

Supplementary Figure 19

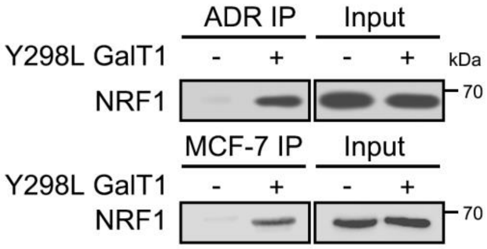

Supplementary Figure 19

The O-GlcNAc enzymatic labeling system was employed to confirm the O-GlcNAc modification of endogenous NRF1 in MCF-7 and ADR cells. ADR and MCF-7 cell lysates were incubated with Y289L GalT1 and UDP-GalNAz and then subjected to click reaction with alkyne-biotin. Streptavidin beads were used to capture O-GlcNAc proteins. An anti-NRF1 antibody was used for immunoblotting. All blots are representative of at least two biologically independent experiments. Source Data are provided as a Source Data file.

Supplementary Figure 20

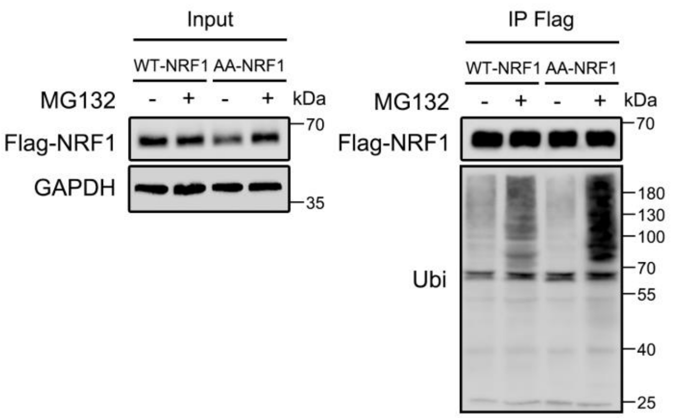

Supplementary Figure 20

O-GlcNAc regulates the stability of NRF1 through ubiquitin-dependent degradation. ADR cells stably expressing Flag-WT-NRF1 or Flag-AA-NRF1 were treated with 10 μM MG132 for 10 h. NRF1 immunoprecipitation was performed using anti-Flag magnetic beads, and immunoprecipitated fractions were analyzed by immunoblotting for ubiquitin (Ubi). All blots are representative of at least two biologically independent experiments. Source Data are provided as a Source Data file.

Supplementary Figure 21

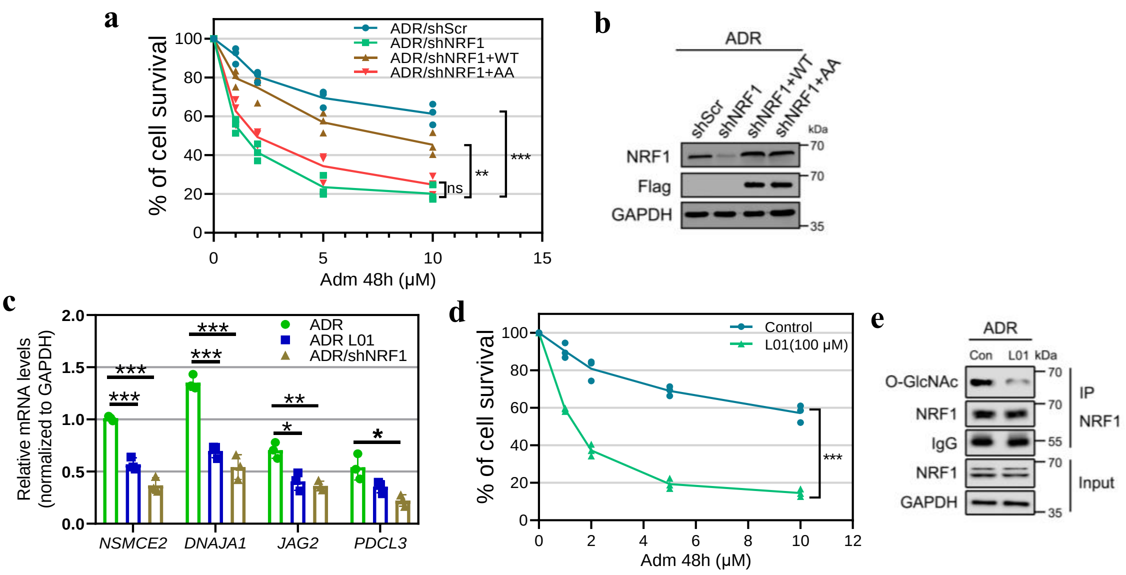

Supplementary Figure 21

O-GlcNAc NRF1 plays a key role in genotoxic adaption and downstream gene expression. (**a**) NRF1 knockdown significantly increased Adm genotoxic effect in ADR cells. ADR cells were stably transfected with NRF1 shRNA (ADR/shNRF1) or scrambled shRNA (ADR/shScr). The rescue expression of Flag-WT-NRF1 and Flag-AA-NRF1 were further performed in ADR/shNRF1 cells (ADR/shNRF1+WT and ADR/shNRF1+AA, respectively). Cells were subsequently treated with increasing doses of Adm for 48 h. The cell viability was assessed using a CCK8 assay. Replicates are represented, n = 3 biologically independent experiments, $**p < 0.01$, $***p < 0.001$ (two-sided unpaired Student's t-test). *p* values: 0.00044 (ADR/shScr vs. ADR/shNRF1), 0.00334 (ADR/shNRF1 vs. ADR/shNRF1+WT), 0.235895 (ADR/shNRF1 vs. ADR/shNRF1+AA). Experiments were repeated independently two times with similar results. (**b**) The protein levels of NRF1 were analyzed by immunoblotting. All blots are representative of at least two biologically independent experiments. (**c**) ADR cells were treated with 100 µM L01 for 48 h. The transcript levels of NRF1 target gene (*NSMCE2*, *DNAJA1*, *JAG2* and *PDCL3*) were analyzed by qPCR in ADR and ADR/shNRF1 cells. The data are presented as the means ± SEM., n = 3 biologically independent experiments, $*p < 0.05$, $**p < 0.01$, $***p < 0.001$ (two-sided unpaired Student's t-test,). *p* value: 0.000316 (ADR vs. ADR L01, *NSMCE2*), 0.000273 (ADR vs. ADR L01,

DNAJA1), 0.011432 (ADR vs. ADR L01, *JAG2*), 0.000138 (ADR vs. ADR/shNRF1, , *NSMCE2*), 0.000531 (ADR vs. ADR/NRF1, *DNAJA1*), 0.00284 (ADR vs. ADR/NRF1, *JAG2*), 0.014592 (ADR vs. ADR/NRF1, *PDCL3*). Experiments were repeated independently two times with similar results. (**d**) ADR cells were treated with 100 μM L01 and increasing doses of Adm for 48 h. The cell viability was assessed using a CCK8 assay. Replicates are represented, n = 3 biologically independent experiments, ***$p < 0.001$ (two-sided unpaired Student's t-test). *p* values: 0.000126. Experiments were repeated independently two times with similar results. (**e**) With the treatment of 100 μM L01 for 48 h, endogenic NRF1 was immunoprecipitated in ADR cells. O-GlcNAc modification of NRF1 was analyzed by immunoblotting (CTD110.6). All blots are representative of at least two biologically independent experiments. (**a**-**e**) Source Data are provided as a Source Data file.

Supplementary Figure 22

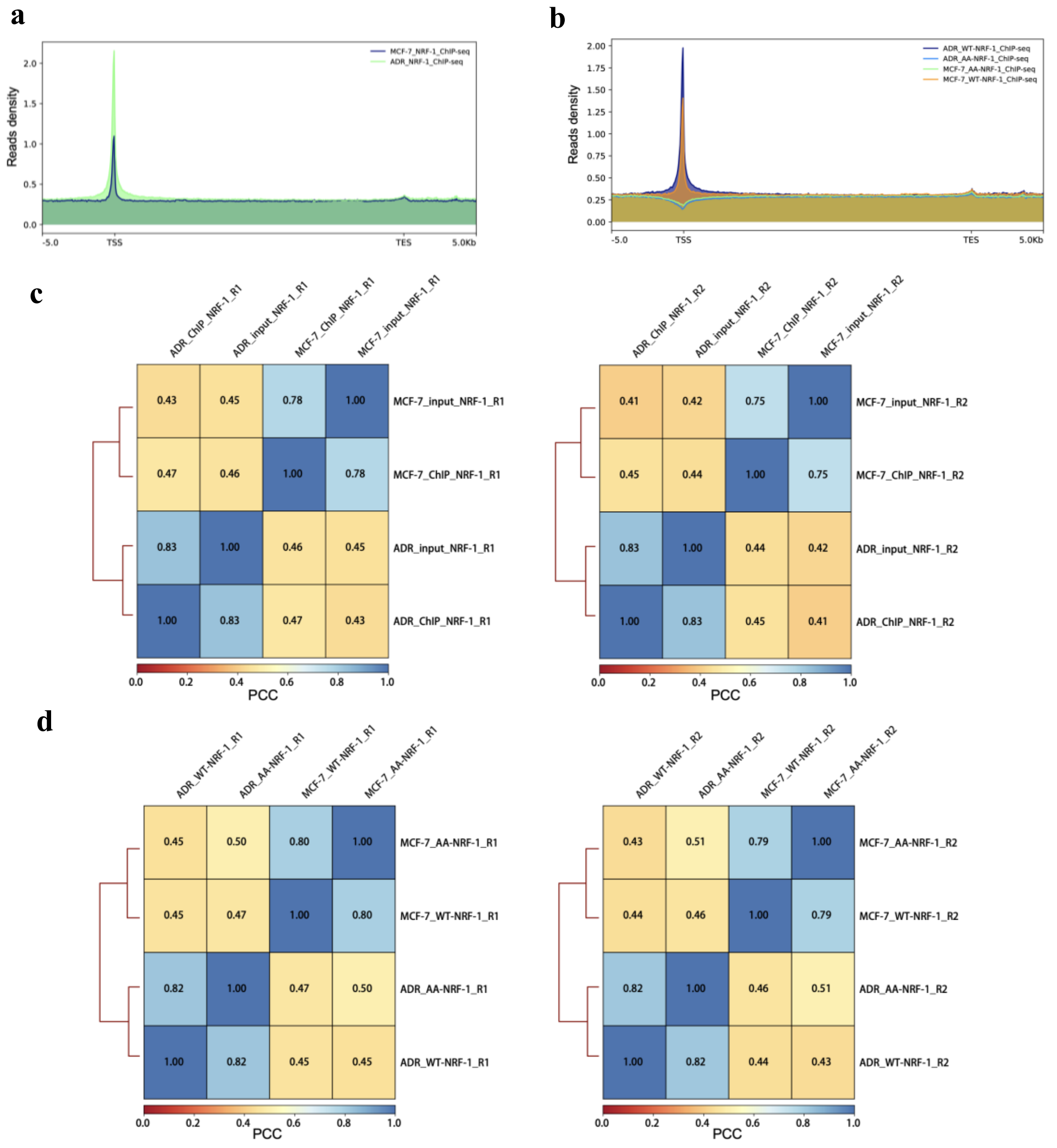

Supplementary Figure 22

Bioinformatics analysis of MCF-7 and ADR NRF-1 ChIP-seq datasets. (**a**) Image shows the average enrichment profiles of MCF-7 and ADR NRF-1 ChIP-seq reads at all gene regions in the reference genome hg19. The TSSs and TESs are indicated. (**b**) Image shows the average enrichment profiles of MCF-7 WT-NRF-1, MCF-7 AA-NRF-1, ADR WT-NRF-1 and ADR AA-NRF-1 ChIP-seq reads at all gene regions in the reference genome hg19. The TSSs and TESs are indicated. (**c**) Correlation between NRF-1 ChIP-seq and the input datasets in MCF-7 and ADR cells. (**d**) Correlation between WT-NRF-1 and AA-NRF-1 ChIP-seq datasets in MCF-7 and ADR cells. For (**c**) and (**d**), two biological repetitive tests (R1 and R2) are indicated. Heat maps were

generated by global Pearson correlation analysis of genome-wide reads from each bam file. The PCC is indicated.

Supplementary Figure 23

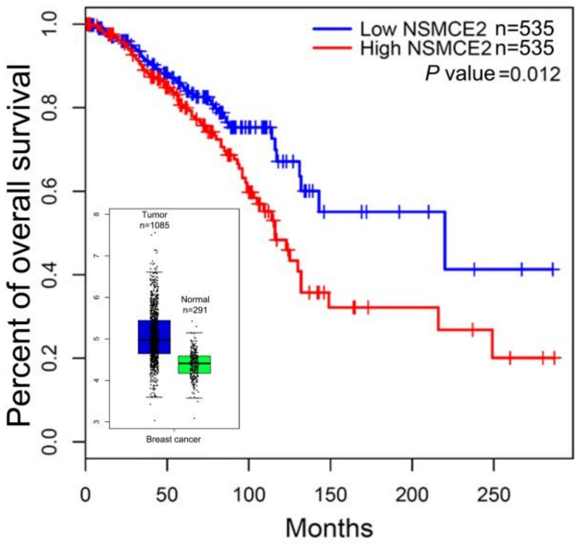

Supplementary Figure 23

Kaplan-Meier curve showing the difference in overall survival between high and low *NSMCE2* expression in breast cancer patient data from the Gene Expression Profiling Interactive Analysis (GEPIA) database [http://gepia.cancer-pku.cn/index.html]. High NSMCE2 samples (n = 535 independent patient samples) with expression levels higher than the median of transcripts of per million (TPM) comprise the high-expression cohort. Low *NSMCE2* samples (n = 535 independent patient samples) with expression level lower than the median of TPM formed the low-expression cohort. The *P* value was calculated using log-rank Mantel-Cox test. The expression levels of *NSMCE2* in breast cancer and normal tissues based on the GEPIA database are also shown. Tumor samples n = 1085 independent patient samples. Normal samples n = 291 independent healthy people samples.
