# Supplementary material for: Proteomic profiling and genome-wide mapping of O-GlcNAc chromatin-associated proteins reveal an O-GlcNAc-regulated genotoxic stress response
Source: Nat Commun. 2020 Nov 19;11:5898. doi: 10.1038/s41467-020-19579-y (PMC7678849; doi:10.1038/s41467-020-19579-y)
Supplement: Supplementary file 3 — Description of Additional Supplementary Files [file 41467_2020_19579_MOESM3_ESM.docx]

Description of additional Supplementary Data flies

File Name: Supplementary Data 1

Description: O-GlcNAz proteins identified from the chromatin of MCF-7 and ADR cells by LC-MS/MS.

File Name: Supplementary Data 2

Description: OCTFs identified from the chromatin of MCF-7 and ADR cells by LC-MS/MS. The TF family and Biological Process (GO) of these OCTFs were indicated.

File Name: Supplementary Data 3

Description: 7112 DEGs identifed by RNA-seq (MCF-7 vs ADR).

File Name: Supplementary Data 4

Description: Quantitative proteomics comparisons between MCF-7 and ADR O-GlcNAz chromatin.

File Name: Supplementary Data 5

Description: Differential quantitative OCTFs in LC-MS/MS.

File Name: Supplementary Data 6

Description: Quantitative proteomics comparisons between MCF-7 and ADR whole-cell proteins.

File Name: Supplementary Data 7

Description: COGC-seq peaks in MCF-7 and ADR cells.

File Name: Supplementary Data 8

Description: sWGA lectin ChIP-seq peaks in MCF-7 and ADR cells.

File Name: Supplementary Data 9

Description: Differential quantitative COGC-seq peaks between MCF-7 and ADR cells using Manorm and Diffbind software.

File Name: Supplementary Data 10

Description: Motif enrichment analysis of TFs binding sites and targeting genes using MEME suite.

File Name: Supplementary Data 11

Description: Motif enrichment analysis in ADR and MCF-7 COGC-seq peaks using MEME suite.

File Name: Supplementary Data 12

Description: Motif enrichment analysis in differential quantitative COGC-seq peaks (MAnorm) using MEME suite.

File Name: Supplementary Data 13

Description: Putative TFs identified in the COGC-seq peaks overlapping with OCTFs identified from the proteomics analysis. The overlapping OCTF-targeting genes were also shown.

File Name: Supplementary Data 14

Description: Predicted regulatory network between genotoxic stress responsive OCTFs and the corresponding downstream DEGs in MCF-7 and ADR cells.

File Name: Supplementary Data 15

Description: NRF-1 uniquely bound sites in ADR cells.

File Name: Supplementary Data 16

Description: PCR promoters used in this study.
