# Supplementary material for: Proteomic profiling and genome-wide mapping of O-GlcNAc chromatin-associated proteins reveal an O-GlcNAc-regulated genotoxic stress response
Source: Nat Commun. 2020 Nov 19;11:5898. doi: 10.1038/s41467-020-19579-y (PMC7678849; doi:10.1038/s41467-020-19579-y)
Supplement: Supplementary file 20 — Reporting Summary [file 41467_2020_19579_MOESM20_ESM.pdf]

nature research

Corresponding author(s): Jianing Zhang
Yan Ren

Last updated by author(s): Oct 9, 2020

Reporting Summary

Nature Research wishes to improve the reproducibility of the work that we publish. This form provides structure for consistency and transparency in reporting. For further information on Nature Research policies, see Authors & Referees and the Editorial Policy Checklist .

Statistics

For all statistical analyses, confirm that the following items are present in the figure legend, table legend, main text, or Methods section.

n/a	Confirmed	
☐	☒	The exact sample size (*n*) for each experimental group/condition, given as a discrete number and unit of measurement
☐	☒	A statement on whether measurements were taken from distinct samples or whether the same sample was measured repeatedly
☐	☒	The statistical test(s) used AND whether they are one- or two-sided<br>*Only common tests should be described solely by name; describe more complex techniques in the Methods section.*
☒	☐	A description of all covariates tested
☒	☐	A description of any assumptions or corrections, such as tests of normality and adjustment for multiple comparisons
☐	☒	A full description of the statistical parameters including central tendency (e.g. means) or other basic estimates (e.g. regression coefficient) AND variation (e.g. standard deviation) or associated estimates of uncertainty (e.g. confidence intervals)
☐	☒	For null hypothesis testing, the test statistic (e.g. *F*, *t*, *r*) with confidence intervals, effect sizes, degrees of freedom and *P* value noted<br>*Give P values as exact values whenever suitable.*
☒	☐	For Bayesian analysis, information on the choice of priors and Markov chain Monte Carlo settings
☒	☐	For hierarchical and complex designs, identification of the appropriate level for tests and full reporting of outcomes
☒	☐	Estimates of effect sizes (e.g. Cohen's *d*, Pearson's *r*), indicating how they were calculated

Our web collection on statistics for biologists contains articles on many of the points above.

Software and code

Policy information about availability of computer code

Data collection	No samples were excluded for any analysis.<br>The absorbance was collected using Gen5 software (v5.0).<br>For qPCR, data were collected with Multiplate RQ software (v1.00).<br>For western blotting, images were collected with AlphaView software v2.10 (Protein Sample).<br>For LC-MS/MS analysis, raw data were collected using Xcalibur software (v2.0.57).<br>For next generation sequencing, raw reads were collected using BGISEQ-500 software (v 0.3.8.1).
Data analysis	In general, results presented as mean ± SEM were collected and calculated using Microsoft Excel 2019 and GraphPad Prism (v8.0).<br>COGC-seq, ChIP-seq and RNA-seq base calling was conducted using BGISEQ-500 software (v0.3.8.1) BGISEQ-500 software (v 0.3.8.1).<br>For next generation sequencing, The raw datasets were analyzed using FastQC (v 0.11.8), Samtools (v 1.2), Bowtie2 (v 2.3.4.3), Bedtools (v2.27.1), MACS (v 2.1.1), Integrative Genomics Viewer (IGV; v2.3.4), deeptools (v 3.3.2.0.0), Metascape (v 3.0), Cytoscape (v 3.6.1), MEME (v 5.0.1), DiffBind (v 2.10.0), MAnorm (v 1.2.0), Homer (v 4.11), SOAPnuke (v 2.1.0), HISAT2 (v 2.1.0), RSEM (v 1.2.4), ChIPseeker (v 1.18.0); DESeq2 (v 1.18.1).<br>For LC-MS/MS analysis, MaxQuant (v 1.5.3.28), STRING software (v 11.0), Metascape software (v 3.0) were used.<br>Other bioinformatic analyses were performed using the phyper function in the R software package (v3.4.3), R-package: beanplot (v 1.2), R-package: ggplot2 (v 3.0.0), R-package: igraph (v 1.2.1), R-package: venneuler (v 1.1-0).

For manuscripts utilizing custom algorithms or software that are central to the research but not yet described in published literature, software must be made available to editors/reviewers. We strongly encourage code deposition in a community repository (e.g. GitHub). See the Nature Research guidelines for submitting code & software for further information.

Data

Policy information about availability of data

All manuscripts must include a data availability statement. This statement should provide the following information, where applicable:

- Accession codes, unique identifiers, or web links for publicly available datasets
- A list of figures that have associated raw data
- A description of any restrictions on data availability

The raw data of COGC-seq, NRF1 ChIP-seq and RNA-seq is available in the Gene Expression Omnibus database under the accession number GSE141698 [https://www.ncbi.nlm.nih.gov/geo/query/acc.cgi?acc=GSE141698].
The raw mass spectral data in our study is available via iProX with identifier PXD016713 [https://www.iprox.org//page/project.html?id=IPX0001893000].
The other published ChIP-seq and other sequencing raw data used in this study is available in the Gene Expression Omnibus database: MCF-7 cells ChIP-seq H3K27ac, H3K4me3 (GSE97481, [https://www.ncbi.nlm.nih.gov/geo/query/acc.cgi?acc=GSE97481]), H3K27me3 (GSE96363, [https://www.ncbi.nlm.nih.gov/geo/query/acc.cgi?acc=GSE96363]) and H3K4me1 (GSE86714, [https://www.ncbi.nlm.nih.gov/geo/query/acc.cgi?acc=GSE86714]), HCF-1 (GSE91992 [https://www.ncbi.nlm.nih.gov/geo/query/acc.cgi?acc=GSE91992]), SP1 (GSE92014, [https://www.ncbi.nlm.nih.gov/geo/query/acc.cgi?acc=GSE92014]), NRF1 (GSE91522, [https://www.ncbi.nlm.nih.gov/geo/query/acc.cgi?acc=GSE91522]), HEK293 cells OGT, O-GlcNAc ChIP-seq (GSE36620 [https://www.ncbi.nlm.nih.gov/geo/query/acc.cgi?acc=GSE36620]), BJAB cells O-GlcNAc ChIP-seq (GSE86154 [https://www.ncbi.nlm.nih.gov/geo/query/acc.cgi?acc=GSE86154]), MCF-7 cells RNA-Pol II ChIP-seq (GSE34001 [https://www.ncbi.nlm.nih.gov/geo/query/acc.cgi?acc=GSE34001]) and MCF-7 GRO-seq (GSE96859 [https://www.ncbi.nlm.nih.gov/geo/query/acc.cgi?acc=GSE96859]).
The raw data underlying Figure 1b-c, 2a, 4e, 5c, 5e-f, 6a, 6d-i, 7d, 8a-d, as well as Supplementary Figure 1a-b, 1d, 2, 3, 4, 6c, 7e, 9a, 11a, 12a-b, 13a-c, 14b, 15a, 16a, 17a-e, 19, 20, 21a-e are available in the Source Data file. All other data supporting the findings of this study are available from the corresponding authors upon reasonable request.

Field-specific reporting

Please select the one below that is the best fit for your research. If you are not sure, read the appropriate sections before making your selection.

- [x] Life sciences
- [ ] Behavioural & social sciences
- [ ] Ecological, evolutionary & environmental sciences

For a reference copy of the document with all sections, see nature.com/documents/nr-reporting-summary-flat.pdf

Life sciences study design

All studies must disclose on these points even when the disclosure is negative.

Sample size

We did not perform any patient or in-vivo analysis as all data was generated using cell-culture standards. No statistical method was used to predetermine sample size. Because it is not a population study and the important issue is that the biological samples are stress adapted for comparison, not the sample size. n = 3 biologically independent experiments were used this study. This information is provided in all figure legends.

Data exclusions

No data were excluded from these assays.

Replication

Experiments were performed at minimum of two times. Two biologically independent replicates were performed in COGC-seq, ChIP-seq and RNA-seq experiments. sWGA lectin ChIP-seq was performed once. Nine biological replicates of the proteomic analysis were performed. The crystal violet staining results were reproduced in two biologically independent experiments. Other experiments were performed at least three times (biological replicates) to obtain statistical significance. Experiments were repeated independently two times with similar results. All blots are representative of at least two biologically independent experiments. This information is provided in all figure legends. All replications were successful.

Randomization

Our study did not involve patients and therefore we did not use trial and treatment groups for the analysis.

Blinding

Our study did not involve patients and therefore blinding trials were not used. The investigators were blinded to RNA-seq, ChIP-seq, COGC-seq and LC-MS/MS analysis, and all other data collection and/or analysis.

Reporting for specific materials, systems and methods

We require information from authors about some types of materials, experimental systems and methods used in many studies. Here, indicate whether each material, system or method listed is relevant to your study. If you are not sure if a list item applies to your research, read the appropriate section before selecting a response.

Materials & experimental systems

n/a	Involved in the study
	x Antibodies
	x Eukaryotic cell lines
x	Palaeontology
x	Animals and other organisms
x	Human research participants
x	Clinical data

Methods

n/a	Involved in the study
	x ChIP-seq
x	Flow cytometry
x	MRI-based neuroimaging

Antibodies

Antibodies used

For western blot or lectin blot:

The primary antibodies used were anti-O-GlcNAc CTD110.6 (BioLegend, #838004, 1:1,000), anti-O-GlcNAc RL2 (Abcam, #ab93858, 1:1,000), anti-SP1 (Abcam, #ab13370, 1:1,000), anti-KLF5 (CST, #51586, 1:1,000), anti-NFKB1 (CST, #13586, 1:1,000), anti-JUN (Proteintech, #24909-1-AP, 1:500), anti-NRF1 (CST, #46743, 1:1,000), anti-OGT (Proteintech, #11576-2-AP, 1:1,000), anti-GAPDH (CST, #5174, 1:1,000), anti-HCF-1 (CST, #50708, 1:1,000), anti-Flag (CST, #14793, 1:1,000), anti-Histone 3 (CST, #4499, 1:1,000), anti-NSMCE2 (Abcam, #ab241564, 1:1,000), anti-Ubi (Abcam, #ab134953, 1:2,000). Lectin sWGA (Vector Laboratories, #B-1025S, 1:2,000) was used for lectin blotting. The appropriate secondary antibody used were anti-mouse IgG-HRP (CST, # 7076, 1:20,000), anti-rabbit IgG-HRP (CST, #7074, 1:20,000), anti-mouse IgM-HRP (Abcam, #ab97230, 1:20,000), Streptavidin-HRP (CST, #3999, 1:50,000).

For co-immunoprecipitation:

The antibodies and beads used for IP were anti-SP1 (Abcam, #ab13370, 1:100), anti-KLF5 (CST, #51586, 1:100), anti-JUN (Proteintech, #24909-1-AP, 1:50), anti-NRF1 (CST, #46743, 1:100), and anti-Flag-magnetic beads (Bimake, # B26102).

Non-immune IgG (#sc-2027, Santa Cruz), anti-SP1 (Abcam, #ab13370, 1:10), anti-KLF5 (CST, #51586, 1:10), anti-NRF1 (CST, #46743, 1:10) and anti-Flag-magnetic beads (Bimake, 100 μL) were used for ChIP.

Streptavidin-magnetic beads (BEAVER Life Science, #22308-10) was used for COGC-seq.

sWGA-agarose beads (Vector Laboratories, #AL-1023S) was used for sWGA lectin ChIP-seq.

Validation

Commercially available antibodies used were validated by their respective manufactures' catalogue files to be compatible.

anti-O-GlcNAc CTD110.6 (BioLegend, #838004, 1:1,000, western blot): validation data provided by the supplier (https://www.biolegend.com/en-us/products/purified-anti-o-glcnac-antibody-13607) and also characterized in detail in Dias WB, et al. 2009. J. Biol. Chem. 32:21327.

anti-O-GlcNAc RL2 (Abcam, #ab93858, 1:1,000, western blot): validation data provided by the supplier (https://www.abcam.cn/rl2-antibody-ab93858.html) and also characterized in detail in Han C, et al. 2017, Nat Commun 8:1491.

anti-SP1 (Abcam, #ab13370, 1:1,000 for western blot, 1:100 for co-immunoprecipitation, 1:10 for ChIP): validation data provided by the supplier (https://www.abcam.cn/sp1-antibody-ab13370.html) and validated in our lab by siRNA knockdown.

anti-KLF5 (CST, #51586, 1:1,000 for western blot, 1:100 for co-immunoprecipitation, 1:10 for ChIP): validation data provided by the supplier (https://www.cellsignal.cn/products/primary-antibodies/klf5-d7s3f-rabbit-mab/51586?N=4294956287&Ntt=%2351586&fromPage=plp) and validated in our lab by siRNA knockdown.

anti-NFKB1 (CST, #13586, 1:1,000, western blot): validation data provided by the supplier (https://www.cellsignal.cn/products/primary-antibodies/nf-kb1-p105-p50-d4p4d-rabbit-mab/13586?N=4294956287&Ntt=%2313586&fromPage=plp) and also characterized in detail in Orian, A. et al. 2000, EMBO J 19, 2580-91.

anti-JUN (Proteintech, #24909-1-AP, 1:500 for western blot, 1:50 for co-immunoprecipitation): validation data provided by the supplier (https://www.ptgcn.com/Products/JUN-Antibody-24909-1-AP.htm).

anti-NRF1 (CST, #46743, 1:1,000 for western blot, 1:100 for co-immunoprecipitation, 1:10 for ChIP): validation data provided by the supplier (https://www.cellsignal.cn/products/primary-antibodies/nrf1-d9k6p-rabbit-mab/46743?N=4294956287&Ntt=%2346743&fromPage=plp) and validated in our lab by shRNA knockdown.

anti-OGT (Proteintech, #11576-2-AP, 1:1,000, western blot): validation data provided by the supplier (https://www.ptgcn.com/Products/OGT-Antibody-11576-2-AP.htm).

anti-GAPDH (CST, #5174, 1:1,000, western blot): validation data provided by the supplier (https://www.cellsignal.cn/products/primary-antibodies/gapdh-d16h11-xp-rabbit-mab/5174?N=4294956287&Ntt=%235174&fromPage=plp) and also characterized in detail in Zheng, L. et al. 2003, Cell 114, 255-66.

anti-HCF-1 (CST, #50708, 1:1,000, western blot): validation data provided by the supplier (https://www.cellsignal.cn/products/primary-antibodies/hcfc1-antibody-carboxy-terminal-antigen/50708?N=4294956287&Ntt=%2350708&fromPage=plp) and also characterized in detail in Lazarus, M.B. et al. 2013, Science 342, 1235-9.

anti-Flag (CST, #14793, 1:1,000, western blot): validation data provided by the supplier (https://www.cellsignal.cn/products/primary-antibodies/dykddddk-tag-d6w5b-rabbit-mab-binds-to-same-epitope-as-sigma-s-anti-flag-m2-antibody/14793?N=4294956287&Ntt=%2314793&fromPage=plp).

anti-Histone 3 (CST, #4499, 1:1,000, western blot): validation data provided by the supplier (https://www.cellsignal.cn/products/primary-antibodies/histone-h3-d1h2-xp-rabbit-mab/4499?N=4294956287&Ntt=%234499&fromPage=plp) and also characterized in detail in Dai J et al. 2005, Genes Dev 19, 472–88.

anti-NSMCE2 (Abcam, #ab241564, 1:1,000, western blot): validation data provided by the supplier (https://www.abcam.cn/mms21-antibody-215c-ab241564.html) and validated in our lab by siRNA knockdown.

anti-Ubi (Abcam, #ab134953, 1:2,000, western blot): validation data provided by the supplier (https://www.abcam.cn/ubiquitin-antibody-epr8830-ab134953.html) and also characterized in detail in Lynch JM et al. 2018, J Biol Chem 293:20137-20156.

Lectin sWGA (Vector Laboratories, #B-1025S, 1:2,000, lectin blot): validation data provided by the supplier (https://vectorlabs.com/biotinylated-succinylated-wheat-germ-agglutinin-wga.html).
The availability of Streptavidin-magnetic beads (BEAVER Life Science, #22308-10) and sWGA-agarose beads (Vector Laboratories, #AL-1023S) were confirmed in a previous report (Liu et al., Nature Chemical Biology 2017, 13(2): 161-167). To further validate Streptavidin-magnetic beads and sWGA-agarose beads for ChIP-seq-like experiments, we performed immunoprecipitation of formaldehyde cross-linked cells that with/without O-GlcNAc inhibitor L01, followed by western blot.

Eukaryotic cell lines

Policy information about cell lines

Cell line source(s)
MCF-7, MDA-MB-231, K562, HCT-15 and HEK 293T (293T) cells were obtained from Type Culture Collection of the Chinese Academy of Sciences (Shanghai, China) and were used within 6 months from resuscitation.
Adm (Adriamycin) or Fu (Fluorouracil) was added to cell cultures in stepwise increasing concentrations from 0.1 to 10 μM for 4 months to develop an ADR or FU subline, namely MCF-7/ADR, K562/ADR and HCT-15/FU, correspondingly.

Authentication
All cell lines used were STR tested for identity.

Mycoplasma contamination
All cells were routinely tested for mycoplasma and confirmed negative.

Commonly misidentified lines (See ICLAC register)
No commonly misidentified cell lines were used in this study.

ChIP-seq

Data deposition

☒ Confirm that both raw and final processed data have been deposited in a public database such as GEO.

☒ Confirm that you have deposited or provided access to graph files (e.g. BED files) for the called peaks.

Data access links
May remain private before publication.
GEO accession number for data generated in this study is GSE141698.
Link:
https://www.ncbi.nlm.nih.gov/geo/query/acc.cgi?acc=GSE141698

Files in database submission

GSM No.	processed data file	raw file
GSM4211228	COGC-seq_ADR_Input_R1.bw	COGC-seq_ADR Input_R1.fq
GSM4211229	COGC-seq_ADR_Input_R2.bw	COGC-seq_ADR_Input_R2.fq
GSM4211230	COGC-seq_MCF-7_Input_R1.bw	COGC-seq_MCF-7_Input_R1.fq
GSM4211231	COGC-seq_MCF-7_Input_R2.bw	COGC-seq_MCF-7_Input_R2.fq
GSM4211232	COGC-seq_ADR_R1.bw	COGC-seq_ADR_R1.fq
GSM4211233	COGC-seq_ADR_R2.bw	COGC-seq_ADR_R2.fq
GSM4211234	COGC-seq_MCF-7_R1.bw	COGC-seq-MCF-7_R1.fq
GSM4211235	COGC-seq_MCF-7_R2.bw	COGC-seq-MCF-7_R2.fq
GSM4211236	sWGA_ADR_Input.bw	sWGA_ADR_Input.fq
GSM4211237	sWGA_MCF-7_Input.bw	sWGA_MCF-7_Input.fq
GSM4211238	sWGA_ADR_IP.bw	sWGA_ADR.fq
GSM4211239	sWGA_MCF-7_IP.bw	sWGA_MCF-7.fq
GSM4766285	ChIP-seq_ADR_Flag-AA-NRF-1_R1.bw	ChIP-seq_ADR_Flag-AA-NRF-1_R1.fq
GSM4766286	ChIP-seq_ADR_Flag-AA-NRF-1_R2.bw	ChIP-seq_ADR_Flag-AA-NRF-1_R2.fq
GSM4766287	ChIP-seq_ADR_Flag-WT-NRF-1_R1.bw	ChIP-seq_ADR_Flag-WT-NRF-1_R1.fq
GSM4766288	ChIP-seq_ADR_Flag-WT-NRF-1_R2.bw	ChIP-seq_ADR_Flag-WT-NRF-1_R2.fq
GSM4766289	ChIP-seq_ADR_input_Flag-AA-NRF-1_R1.bw	ChIP-seq_ADR_input_Flag-AA-NRF-1_R1.fq
GSM4766290	ChIP-seq_ADR_input_Flag-AA-NRF-1_R2.bw	ChIP-seq_ADR_input_Flag-AA-NRF-1_R2.fq
GSM4766291	ChIP-seq_ADR_input_Flag-WT-NRF-1_R1.bw	ChIP-seq_ADR_input_Flag-WT-NRF-1_R1.fq
GSM4766292	ChIP-seq_ADR_input_Flag-WT-NRF-1_R2.bw	ChIP-seq_ADR_input_Flag-WT-NRF-1_R2.fq
GSM4766293	ChIP-seq_ADR_input_NRF-1_R1.bw	ChIP-seq_ADR_input_NRF-1_R1.fq
GSM4766294	ChIP-seq_ADR_input_NRF-1_R2.bw	ChIP-seq_ADR_input_NRF-1_R2.fq
GSM4766295	ChIP-seq_ADR_NRF-1_R1.bw	ChIP-seq_ADR_NRF-1_R1.fq
GSM4766296	ChIP-seq_ADR_NRF-1_R2.bw	ChIP-seq_ADR_NRF-1_R2.fq
GSM4766297	ChIP-seq_MCF-7_Flag-AA-NRF-1_R1.bw	ChIP-seq_MCF-7_Flag-AA-NRF-1_R1.fq
GSM4766298	ChIP-seq_MCF-7_Flag-AA-NRF-1_R2.bw	ChIP-seq_MCF-7_Flag-AA-NRF-1_R2.fq
GSM4766299	ChIP-seq_MCF-7_Flag-WT-NRF-1_R1.bw	ChIP-seq_MCF-7_Flag-WT-NRF-1_R1.fq
GSM4766300	ChIP-seq_MCF-7_Flag-WT-NRF-1_R2.bw	ChIP-seq_MCF-7_Flag-WT-NRF-1_R2.fq
GSM4766301	ChIP-seq_MCF-7_input_Flag-AA-NRF-1_R1.bw	ChIP-seq_MCF-7_input_Flag-AA-NRF-1_R1.fq
GSM4766302	ChIP-seq_MCF-7_input_Flag-AA-NRF-1_R2.bw	ChIP-seq_MCF-7_input_Flag-AA-NRF-1_R2.fq
GSM4766303	ChIP-seq_MCF-7_input_Flag-WT-NRF-1_R1.bw	ChIP-seq_MCF-7_input_Flag-WT-NRF-1_R1.fq
GSM4766304	ChIP-seq_MCF-7_input_Flag-WT-NRF-1_R2.bw	ChIP-seq_MCF-7_input_Flag-WT-NRF-1_R2.fq

GSM4766305	ChIP-seq_MCF-7_input_NRF-1_R1.bw	ChIP-seq_MCF-7_input_NRF-1_R1.fq
GSM4766306	ChIP-seq_MCF-7_input_NRF-1_R2.bw	ChIP-seq_MCF-7_input_NRF-1_R2.fq
GSM4766307	ChIP-seq_MCF-7_NRF-1_R1.bw	ChIP-seq_MCF-7_NRF-1_R1.fq
GSM4766308	ChIP-seq_MCF-7_NRF-1_R2.bw	ChIP-seq_MCF-7_NRF-1_R2.fq
GSM4766309	Diff_RNA-seq_MCF7_ADR_R2.txt	ADR RNA-seq-R2.fq
GSM4766310	RNA-seq_MCF7_ADR_R2.txt	MCF7 RNA-seq-R2.fq
GSM4211240	Diff_RNA-seq_MCF7andADR.txt	ADR RNA-seq.fq
GSM4211241	Diff_RNA-seq_MCF7andADR.txt	MCF-7 RNA-seq.fq

Genome browser session
(e.g. UCSC)

IGV

Methodology

Replicates

For each experiment, 7-8 individual 10 cm dishes of MCF-7 cells or ADR cells were fixed and cell pellet were collected from each dish. The sonicated nuclear extracts were pooled and mixed well. 100 µg sonicated chromatin was used for each ChIP-seq-like experments. sWGA lectin ChIP-seq was performed once. Two biological replicates of the RNA-seq, COGC-seq and other ChIP-seq were done.

Sequencing depth

All single-end reads.

Sample	Clean reads	Mapped reads
COGC-seq ADR Input-R1	85,476,292	82,647,311
COGC-seq ADR Input-R2	89,517,052	85,814,079
COGC-seq MCF_7 Input-R1	85,490,163	82,759,398
COGC-seq MCF-7 Input-R2	89,516,456	85,790,107
COGC-seq ADR-R1	85,504,041	82,435,665
COGC-seq ADR-R2	89,517,550	85,160,863
COGC-seq MCF_7-R1	85,479,583	82,403,186
COGC-seq MCF_7-R2	89,485,341	85,527,551
sWGA-ADR-Input	27,264,143	26,520,664
sWGA-MCF-7-Input	27,349,146	26,521,728
sWGA-ADR IP	27,319,286	26,428,621
sWGA-MCF-7 IP	27,309,005	26,444,588
ChIP-seq_ADR_Flag-AA-NRF-1_R1	21,171,628	20,923,792
ChIP-seq_ADR_Flag-AA-NRF-1_R2	21,197,516	20,945,704
ChIP-seq_ADR_Flag-WT-NRF-1_R1	21,172,369	20,852,883
ChIP-seq_ADR_Flag-WT-NRF-1_R2	21,195,521	20,882,145
ChIP-seq_ADR_input_Flag-AA-NRF-1_R1	21,150,849	20,873,757
ChIP-seq_ADR_input_Flag-AA-NRF-1_R2	21,180,114	20,910,063
ChIP-seq_ADR_input_Flag-WT-NRF-1_R1	21,106,090	20,866,375
ChIP-seq_ADR_input_Flag-WT-NRF-1_R2	21,108,637	20,871,088
ChIP-seq_ADR_input_NRF-1_R1	21,188,148	20,944,.927
ChIP-seq_ADR_input_NRF-1_R2	21,081,028	20,828,125
ChIP-seq_ADR_NRF-1_R1	21,163,591	20,837,584
ChIP-seq_ADR_NRF-1_R2	21,196,401	20,884,141
ChIP-seq_MCF-7_Flag-AA-NRF-1_R1	21,054,482	20,732,598
ChIP-seq_MCF-7_Flag-AA-NRF-1_R2	21,110,978	20,790,141
ChIP-seq_MCF-7_Flag-WT-NRF-1_R1	20,980,564	20,606,423
ChIP-seq_MCF-7_Flag-WT-NRF-1_R2	20,890,898	20,498,759
ChIP-seq_MCF-7_input_Flag-AA-NRF-1_R1	21,160,128	20,772,486
ChIP-seq_MCF-7_input_Flag-AA-NRF-1_R2	21,114,534	20,737,856
ChIP-seq_MCF-7_input_Flag-WT-NRF-1_R1	21,207,904	20,930,831
ChIP-seq_MCF-7_input_Flag-WT-NRF-1_R2	20,993,316	20,697,333
ChIP-seq_MCF-7_input_NRF-1_R1	20,969,246	20,684,237
ChIP-seq_MCF-7_input_NRF-1_R2	21,176,593	20,904,194
ChIP-seq_MCF-7_NRF-1_R1	21,063,983	20,676,620
ChIP-seq_MCF-7_NRF-1_R2	20,977,194	20,613,948
ADR RNA-seq-R2	23,721,397	22,763,053
MCF-7 RNA-seq-R2	23,612,257	22,599,291
ADR RNA-seq	21,682,035	20,339,917
MCF-7 RNA-seq	21,836,111	20,521,577

Antibodies

Streptavidin-magnetic beads (BEAVER Life Science, #22308-10) was used for COGC-seq
sWGA-agarose beads (Vector Laboratories, #AL-1023S) was used for sWGA lectin ChIP-seq
anti-NRF1 (CST, #46743, 1:10), anti-Flag-magnetic beads (Bimake, # B26102) and protein A/G-magnetic beads (Bimake, #B23201) were used for NRF-1, Flag-WT-NRF-1 and Flag-AA-NRF-1 ChIP-seq.

Peak calling parameters	Regions enriched in ChIP-seq-like signal were identified using MACS with corresponding control and parameters -f BAM -g mm -B-p 1e-5 -m 10 30 for narrowPeak calling.
Data quality	All files past fastqc check. Data quality was assessed using MACS by comparing peak enrichment over input controls with a p cutoff value of 1e-5.
Software	FastQC (v 0.11.8);<br>Samtools (v 1.2);<br>Bowtie2 (v 2.3.4.3);<br>Bedtools (v2.27.1);<br>MACS (v 2.1.1);<br>Integrative Genomics Viewer (IGV; v2.3.4)<br>deeptools (v 3.3.2.0.0);<br>Metascape (v 3.0);<br>Cytoscape (v 3.6.1);<br>MEME (v 5.0.1);<br>DiffBind (v 2.10.0);<br>MAnorm (v 1.2.0);<br>Homer (v 4.11);<br>ChIPseeker (v 1.18.0);<br>SOAPnuke (v 2.1.0);<br>HISAT2 (v 2.1.0);<br>RSEM (v 1.2.4);<br>DESeq2 (v 1.18.1);<br>R main package (v 3.4.3);<br>R-package: venneuler (v 1.1-0);<br>R-package: beanplot (v 1.2);<br>R-package: ggplot2 (v 3.0.0);<br>R-package: igraph (v 1.2.1).
